# A High-Throughput Approach to Identify Specific Neurotoxicants / Developmental Toxicants in Human Neuronal Cell Function Assays

**DOI:** 10.14573/altex.1712182

**Published:** 2018-01-21

**Authors:** Johannes Delp, Simon Gutbier, Stefanie Klima, Lisa Hoelting, Kevin Pinto-Gil, Jui-Hua Hsieh, Michael Aichem, Karsten Klein, Falk Schreiber, Raymond R. Tice, Manuel Pastor, Mamta Behl, Marcel Leist

**Affiliations:** 1 In vitro Toxicology and Biomedicine, Dept inaugurated by the Doerenkamp-Zbinden Foundation, University of Konstanz, Konstanz, Germany; 2 Research Training Group RTG1331, University of Konstanz, Konstanz, Germany; 3 Cooperative doctorate college InViTe, University of Konstanz, Konstanz, Germany; 4 Research Program on Biomedical Informatics (GRIB), Dept. of Experimental and Health Sciences, Universitat Pompeu Fabra, Barcelona, Spain; 5 Kelly Government Solutions, Durham, NC, USA; 6 Department of Computer and Information Science, University of Konstanz, Konstanz, Germany; 7 Faculty of Information Technology, Monash University, Melbourne, Australia; 8 Division of National Toxicology Program, National Institute of Environmental Health Sciences, Research Triangle Park, NC, USA

**Keywords:** neurite outgrowth inhibition, cytotoxicity, neurotoxicity, high content imaging, developmental toxicity

## Abstract

The (developmental) neurotoxicity hazard is still unknown for most chemicals. Establishing a test battery covering most of the relevant adverse outcome pathways may close this gap without requiring a huge animal experimentation program. Ideally, each of the assays would cover multiple mechanisms of toxicity. One candidate test is the human LUHMES cell-based NeuriTox test. To evaluate its readiness for larger-scale testing, a proof of concept library, assembled by the U.S. National Toxicology Program (NTP), was screened. Out of the 75 unique compounds, seven were defined as specifically neurotoxic after the hit-confirmation phase and ten further compounds were generally cytotoxic within the concentration range of up to 20 μM. As complementary approach, the library was screened in the PeriTox test, which identifies toxicants affecting the human peripheral nervous system. Of the eight PeriTox hits, five were similar to the NeuriTox hits: rotenone, colchicine, diethylstilbestrol, berberine chloride, and valinomycin. The unique NeuriTox hit, methyl-phenylpyridinium (MPP^+^), is known from *in vivo* studies to affect only dopaminergic neurons (which LUHMES cells are). Conversely, the known peripheral neurotoxicant acrylamide was picked up in the PeriTox, but not in the NeuriTox assay. All of the five common hits had also been identified in the published neural crest migration (cMINC) assay, while none of them emerged as a cardiotoxicant in a previous screen using the same library. These comparative data suggest that complementary *in vitro* tests can pick up a broad range of toxicants, and that multiple test results might help to predict organ specificity patterns.

## 1 Introduction

The transition from method development to actual tests for screening and prioritization is an important advance for *in vitro* toxicology (Crofton et al., 2011; Daneshian et al., 2016; Leist et al., 2008a,b). While this has been achieved in areas like genotoxicity or topical toxicity to skin and eyes (Basketter et al., 2012; Ezendam et al., 2016; Kirkland et al., 2011; Leist et al., 2014; Prinsen et al., 2017), the fields of organ toxicity and developmental toxicity still represent a major challenge (Marx et al., 2016). Especially for neurotoxicity (NT) and developmental neurotoxicity (DNT), multiple tests have been developed, but their comparison using larger compound libraries still lags (Fritsche et al., 2017; Aschner et al., 2017; Smirnova et al., 2014). Developmental neurotoxicity results from gestational or peripartum disturbances of neural cells that eventually lead to an altered connectivity of the neuronal system. For instance, toxicants may inhibit proliferation, differentiation or migration of neural cells (Bal-Price et al., 2015; Aschner et al., 2017). The toxicological manifestation of disturbed key developmental processes is a structural or functional defect of the nervous system.

Current regulatory procedure hardly evaluates DNT for industrial chemicals, while about 100 pesticides have been tested according to OECD TG 426 (OECD, 2007), which requires repeated dosing during pregnancy and lactation (Smirnova et al., 2014; van Thriel et al., 2012; Schmidt et al., 2017; Makris et al., 2009). This test is highly costly (ca. $1–2 million per compound), and its sensitivity has been questioned (Fritsche et al., 2017).

At present, more than 100 compounds (including several drugs) have been found to trigger DNT in animals, while there is strong epidemiological evidence for such effects in humans for only about a dozen compounds (Aschner et al., 2017; Mundy et al., 2015; Grandjean and Landrigan, 2006). The majority of industrial chemicals, and even of drugs, have never been evaluated for DNT (Grandjean and Landrigan, 2006, 2014; Bennett et al., 2016; Crofton et al., 2012). Thus, there is an enormous need for high quality and high throughput *in vitro* testing (Bal-Price et al., 2015; Crofton et al., 2014; Smirnova et al., 2014).

During the last decade, *in vitro* tests have been developed to fill the testing gap (Fritsche et al., 2017; Schmidt et al., 2017). They assess, for instance, the proliferation and differentiation of neuronal precursor cells (Baumann et al., 2016; Culbreth et al., 2012; Fritsche et al., 2005; Hogberg et al., 2010, 2009; Schmuck et al., 2017; Shinde et al., 2017; Balmer et al., 2012), the loss of certain neuronal (sub)populations (Baumann et al., 2016; Culbreth et al., 2012; Pamies et al., 2016; Schmuck et al., 2017; Zimmer et al., 2011b,a), or the impairment of migration (Nyffeler et al., 2017b; Schmuck et al., 2017; Zimmer et al., 2012), neurite outgrowth (Harrill et al., 2011; Krug et al., 2013) or neuronal network formation (Brown et al., 2017; Pamies et al., 2016; Schmuck et al., 2017). Since a single *in vitro* assay cannot cover the complexity of *in vivo* development, plans for test strategies are built on compiling data from a battery of assays that cover all relevant processes (Fritsche et al., 2017; Zimmer et al., 2014).

A cell source of high quality and quantity is a crucial factor for robust tests. One such option is provided by LUHMES cells. They are of human origin and represent extensively characterized neuronal progenitors that can be differentiated within six days into dopaminergic neurons (Smirnova et al., 2016; Krug et al., 2014; Scholz et al., 2011; Schildknecht et al., 2013). In the process of establishing and validating an *in vitro* method, its robustness and suitability for high throughput testing has to be assessed (Leist et al., 2012, 2010). The U.S. National Toxicology Program (NTP) compiled a collection of 80 compounds (herein called NTP80 collection) made up of 75 unique chemicals and internal controls. The focus of this library is on known or suspected DNT/NT compounds as well as compounds of significant interest to the NTP (e.g., flame retardants, PAHs) that have not been tested for DNT/NT activity. This library has been made available for all interested test developers with the vision to generate a comparable data matrix across many DNT and neurotoxicity assays. Initial data on the interference with iPSC differentiation, neurite outgrowth, neural crest cell migration, and cardiotoxicity have been published (Pei et al., 2016; Nyffeler et al., 2017a; Ryan et al., 2016; Sirenko et al., 2017).

In our study, we used the NTP80 collection to evaluate the throughput and quality of the LUHMES cell-based developmental neurotoxicity assay (NeuriTox). The results from the screen were validated in a hit confirmation phase. As follow-up, the library was also screened for peripheral nervous system toxicity (PeriTox assay; Hoelting et al., 2016), and the data were put into context of published screens and of data available from the Tox21 program.

## 2 Material and methods

### Screen library handling

**Fig. 1:**
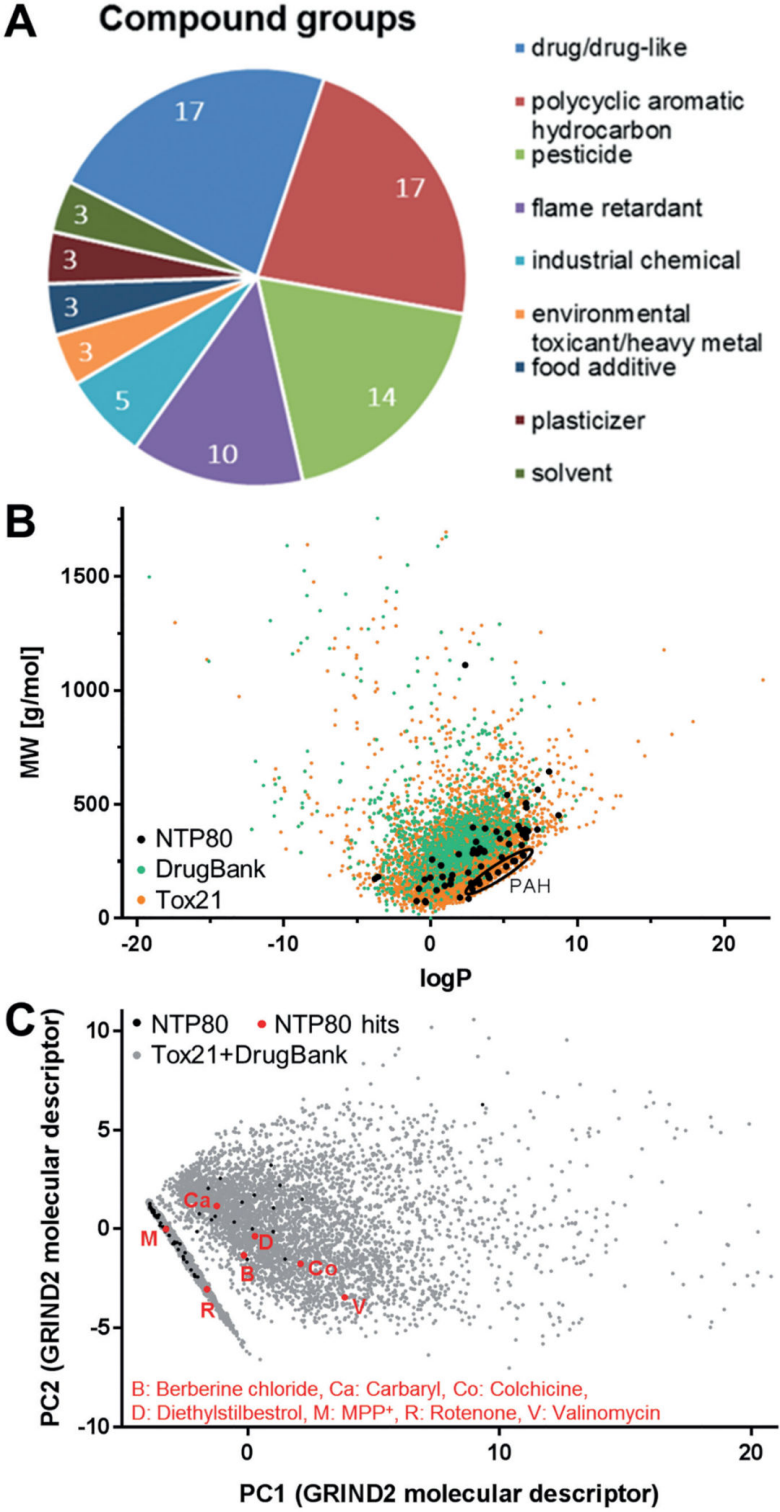
Characterization of the chemical properties of the screened library A) The 75 unique compounds of the NTP80 collection may be classified as drug/drug-like compounds, polycyclic aromatic hydrocarbons, pesticides, flame retardants, industrial chemicals, environmental toxicants, heavy metals, food additives, plasticizers and solvents. The numbers in the circle sectors indicate how many compounds the respective class consists of. B) The molecular weight (MW) of the compounds of the NTP80 collection was plotted against their hydrophobicity (logP). For comparison, the same plot displays the respective data for the Tox21 and DrugBank libraries. For orientation, polycyclic aromatic hydrocarbons (PAH) present in the NTP80 collection were encircled. Detailed data are given in a supplementary file^1^. C) An extensive set of molecular descriptors was generated for the combined Tox21 and DrugBank libraries (grey dots) as described in the methods section. Then, a principal component analysis (PCA) was performed and the first two PCs were used to plot the chemical space of this large compound selection (ca. 9000 compounds) in the background. The same molecular descriptors were then determined for the NTP80 compounds and their positions were marked on the PCA plot (black). The specific hits of the screen described later in this publication are highlighted in red.

The compound library was received as a 96-well “master plate” filled with compounds, i.e., a collection of drug/drug-like compounds, PAH, pesticides, flame retardants, and others (Fig. 1), (Nyffeler et al., 2017a; Ryan et al., 2016; Sirenko et al., 2017). In order to reduce freeze-thaw cycles, save compounds, and test them always after the same number of freeze-thaw cycles, sets of five compounds were transferred from the “master plate” to each “dilution plate” and diluted in DMSO. Subsequently, the dilutions were aliquoted into a “treatment plate” that was equipped with DMSO-solvent controls and narciclasine positive control (50 nM final concentration on cells, Sigma, CAS 2947783–6), sealed, and stored at −80°C until use. This procedure ensured that cells were always treated with 0.1% DMSO (Fig. S3^1^).

### Cell culture and differentiation

For the NeuriTox test (= UKN4), LUHMES (Lund human mesencephalic) cells were characterized and cultured as described in detail earlier (Krug et al., 2013; Lotharius et al., 2005; Scholz et al., 2011). Briefly, cells were maintained in proliferation medium (PM: AdvDMEM/F12 supplemented with 2 mM glutamine, 1x N2 supplement and 40 ng/ml fibroblast growth factor-2) in PLO/fibronectin coated flasks (50 μg/ml poly-L-ornithine (PLO) and 1 μg/ml fibronectin). For differentiation, cells were seeded at a density of 46,000 cells/cm^2^ in PM. After 24 h, they were switched to differentiation medium (DM) containing AdvDMEM/F12 supplemented with 2 mM glutamine, 1x N2 supplement, 2.25 μM tetracycline, 1 mM dibutyryl 3’,5’-cyclic adenosine monophosphate (cAMP), and 2 ng/ml recombinant human glial cell derived neurotrophic factor (GDNF).

The PeriTox test (= UKN5) is based on cells differentiated from the H9 human embryonic stem cell (hESC) line (WA09 line), which was obtained from WiCell (Madison, WI, USA). The import of cells and the experiments were authorized under license no. 170–79-1–4-27 (Robert Koch Institute, Berlin, Germany). The stem cells were cultured according to standard protocols (Thomson et al., 1998) and differentiated into immature dorsal root ganglia neurons, as described previously: after eight days of differentiation with noggin and SB-431542 (dual SMAD inhibition), dorsomorphin (BMP4 signaling inhibitor), DAPT (γ-secretase inhibitor), CHIR (Wnt antagonist) and SU (VEGF, FGF and PDGF signaling inhibitor), the neuronal precursors were cryopreserved for later use in the peripheral neurotoxicity test (PeriTox), which is described in detail in Hoelting et al. (2016).

### Neurotoxicity assays based on neurite outgrowth dynamics

For the NeuriTox test, LUHMES cells were differentiated for 48 h and seeded into 96 well plates at a density of 100,000 cells/cm^2^ in a volume of 90 μl DM without cAMP and GDNF. Treatment was initiated applying 10 μl of a 10x concentrated treatment solution one hour after seeding. At 24 h after treatment, staining mix (SM) was applied (final concentrations: 1 μg/ml H-33342, 1 μM calcein-AM).

For the PeriTox test method, the cells were thawed and seeded at a density of 100,000 cells/cm^2^ in 75 μl PeriTox differentiation medium (PDM) consisting of 25% KSR-S and 75% N2-S media supplemented with 1.5 μM CHIR99021, 1.5 μM SU5402, and 5 μM DAPT on matrigel-coated plates (KSR-S: knockout DMEM with 15% serum replacement, 1 x Glutamax, 1 x nonessential amino acids and 50 mM beta-mercaptoethanol; N2-S: DMEM/F12, with 2 mM Glutamax, 0.1 mg/ml apotransferrin, 1.55 mg/ml glucose, 25 μg/ml insulin, 100 mM putrescine, 30 nM selenium, and 20 nM progesterone). After one hour, 25 μl PDM with 4x concentrated serial dilutions of the test compounds was added to the cells. At 23 h after treatment, the cells were stained with SM and incubated for one additional hour at 37°C.

Image acquisition was performed with an ArrayScan VTI HCS (high content imaging) microscope (Cellomics, Waltham, MA, USA).

### Neurite outgrowth image analysis

The procedure was applied as detailed earlier (Hoelting et al., 2016; Stiegler et al., 2011). After automated imaging, an algorithm was applied that identified the neuronal somata (based on identified nuclei) and subtracted the somatic area from the total neuronal area to obtain the neurite area (NA), i.e., the number of pixels per field covered by neurites. Viability analysis was performed on the same pictures using combined information from H-33342 and calcein channels. Cells with normal-sized nuclei that were calcein-positive were counted as live cells, whereas H-33342 single-positive cells were counted as dead cells. Viability (V) was expressed as percentage of live cells relative to control.

### Data analysis: curve fitting and deriving BMC and EC values

Neurite outgrowth assays were performed at least three times (biological replicates), each run evaluating three technical replicates, i.e., different wells with similar treatment. Neurite area (NA) and viability (V) were always expressed as percentage of the DMSO control. In a first step, matched technical replicates were averaged. Subsequently, these data were averaged across the different experiments. Curve fitting was performed employing a 4-parameter log-logistic function with least squares fit. The upper asymptote of the fit was forced to 100%, the lower asymptote was variable. The variation of DMSO controls was calculated from pooled values of DMSO controls over several experiments.

For calculation of the benchmark concentration values (BMC), a benchmark response (BMR) of three standard deviations of DMSO solvent controls of all assay plates (= “3 x noise level”) was used. EC_50_ concentrations were calculated as the concentration at which the parameter measured (neurite area or viability) declined to 50% of the DMSO-control level. To identify specific effects on neurite outgrowth, the EC_50_ ratio of EC_50_(viability)/EC_50_(neurite area) was calculated. The NeuriTox test system has a specificity threshold ratio of 4, the PeriTox test system has one of 3 (Hoelting et al., 2016; Krug et al., 2013).

In some cases (e.g., non-toxic compounds), no EC_50_ value could be calculated for viability (V). If V was not affected at the highest tested concentration (HTC), then 4 x HTC was used as surrogate EC_50_(V) (marked with ♦; for EC_25_ calculation, the HTC was doubled in this case) (Krug et al., 2013). If the V was affected significantly, but by < 50%, the highest tested concentration was taken as surrogate EC_50_(V) (marked with °). If no EC_50_ for neurite area could be calculated, but neurite outgrowth was inhibited significantly, the highest tested concentration was taken as surrogate EC_50_(NA).

### Analysis of the chemical space

The physicochemical characteristics of the ToxCast + Tox21 (called Tox21), DrugBank, and NTP80 collection chemicals were analyzed as described earlier (Nyffeler et al., 2017a). The structures of the compounds were obtained from the SMILES provided in the original sources, converted to SDFile format (RDKit version 0.9.2^2^) and protonated to pH 7.4 using Moka version 1.1 (Milletti et al., 2007). The molecular weight (MW) and octanol-water distribution coefficient (logP) were obtained using RDKit.

The structures were normalized using standardizer^3^ and converted to 3D using Corina version 3.494 (Sadowski et al., 1994). These were then used to generate GRIND2 descriptors (Pastor et al., 2000; Duran et al., 2009) using Pentacle software version 1.0.6^4^, with default settings. The resulting molecular descriptors were then projected into the principal component analysis (PCA) scores obtained for a collection of ca. 9000 Tox21 and DrugBank compounds (^5^; Wishart et al., 2008) following a similar procedure (supplementary Excel file^6^).

Of the original 75 NTP80 collection compounds, the following four had to be removed from physicochemical analyses because they are salts or contain metallic elements not supported by our methods: (i) methylmercury (II) chloride (MeHgCl), (ii) acetic acid manganese (2+) salt, (iii) bis(tributyltin)oxide and (iv) methylcyclopentadienyl manganese tricarbonyl. For the remaining 71, logP and MW values were obtained. In the process of computing the GRIND2 molecular descriptors, two more compounds had to be removed: saccharin sodium salt hydrate and benzo(b)fluoranthene. Thus, the final series projected in the ToxCast and Tox21 space contained 69 compounds.

### Statistical analysis and data mining

The Tox21 database (retrieved via NTP Sandbox^7^) was mined for all compounds that were active in the NeuriTox test and for which a BMC value could be calculated.

Statistics were performed using GraphPad Prism 5. Neurite outgrowth data were tested for significance by one-way ANOVA followed by a Dunnett’s post-hoc test at the significance level of p < 5%. The summaries displayed are based on independent experiments (different cell lots) unless specified otherwise and are termed “biological replicates”.

## 3 Results and discussion

### 3.1 Characterization of the chemical properties of the screened library

The library (NTP80 collection) used for screening consisted of 75 different compounds of which five were present as independent duplicates. The latter were intended as internal consistency-controls, so that there were “80 compounds” to be tested. The test items were classified into groups according to their main use or chemical structure: drug/drug-like, polycyclic aromatic hydrocarbons (PAH), pesticides and plasticizers made up 72.5% of all compounds. The remaining compounds were environmental and industrial chemicals, as wells as heavy metals (Fig. 1A and Fig. S11). For basic characterization of the library structure, the distribution of physicochemical properties was visualized. A plot of hydrophobicity (logP value) vs molecular weight showed that the library compounds spread out over a sizable part of the plot space defined by other compounds of toxicological concern (here exemplified by ca. 10,000 compounds from the ToxCast/Tox21 and DrugBank databases^5^ (Fig. 1B) (Wishart et al., 2008). However, the NTP80 collection covered only parts of the relevant chemical space, and it was over-represented in the “lower-right part” of the logP-MW plot, where the PAHs clustered.

For broader characterization of the chemical space occupied by the NTP80 collection, a large set of GRIND2 molecular descriptors was calculated for each compound. These descriptors sum up multiple chemical properties and can be considered as a comprehensive approach to characterize a compound. The same descriptors were derived for the compounds of the Tox21 and DrugBank libraries, and the latter data were displayed as a principal component analysis (PCA) scores plot. The NTP80 compounds were then projected into the same PCA scores space (Fig. 1C). Altogether, they were spread out over a sizable area of this PCA scores map. However, it was also clear that larger screens are required to cover the chemical space more comprehensively.

### 3.2 Assay features and performance

**Fig. 2:**
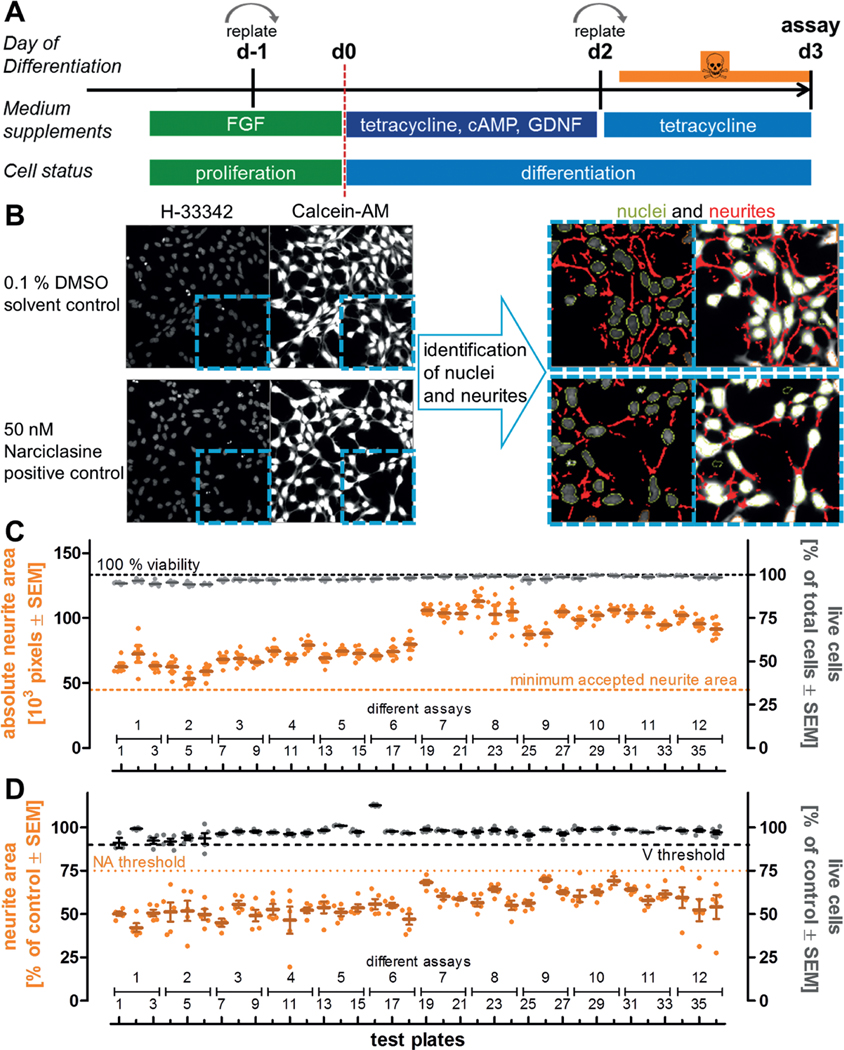
Assay features and performance characteristics for the NeuriTox assay in screening mode A) Differentiation and exposure scheme used to assess neurite outgrowth inhibition of LUHMES cells. The assay was performed from the second (d2) to the third day (d3) of differentiation. To ensure a reproducible start, cells were always replated one day before the start of differentiation (d-1). They were again replated into 96-well plates on d2 and treated 1 h afterwards. Image acquisition was performed 24 h after the start of the treatment. B) Representative images of solvent and positive controls. The nuclei were stained with H-33342 and the cytoplasm was stained with calcein-AM. After automated imaging, an algorithm was applied that recognizes the nuclear area (marked in green) and the overall neuronal area. The program then identified the neuronal somata (based on identified nuclei) and subtracted the somatic area from the neuronal area to obtain the neurite area (marked in red). Viability analysis was performed on the same pictures using combined information from the H-33342 and calcein channels. Cells positive for H-33342 and calcein were counted as live cells, whereas H-33342 single-positive cells were counted as dead cells. Images on the left side have a width of 330 μm. C) To obtain data on overall robustness of the test system, the absolute neurite area and the percentage of live cells were determined for the DMSO solvent control wells of 36 test plates of different days and differentiations. One assay (= biological replicate) consisted of three technical replicate test plates, which had five similar treated DMSO wells each. The data of these five wells are displayed as single points, their mean as a horizontal line. The minimum neurite area to accept a test plate was 45,000 pixels on average per recorded well of untreated cells (orange dotted line). D) Narciclasine (50 nM) was used as positive control to establish screen acceptance standards. Five wells of cells treated with this positive control were included in each assay plate of the screen. Neurite area and viability were measured after 24 h of treatment. Values for neurite area and viability were averaged and normalized against DMSO controls. Data from 5 wells per test plate, 36 test plates and 12 different assays are displayed. The threshold for acceptance of the test plate was a mean neurite area of ≤ 75% of DMSO control, while mean viability had to be ≥ 90%.

In order to run the NeuriTox test in high-throughput mode, proliferating LUHMES cells were switched to medium favoring neuronal differentiation (Fig. 2A), and treated with the compounds. Each potential toxicant was tested at 10 concentrations that were logarithmically spaced. To monitor assay quality, several solvent control (0.1% DMSO) and positive control (50 nM narciclasine) wells were included on each test plate (Fig. S3^1^). After 24 h of exposure, cells were live-stained to assess neurite outgrowth and viability at the same time. LUHMES treated with solvent control (DMSO) grew neurites, which were longer than the diameter of their somata, and the total area covered by neurites within each imaging field was quantified. Cells treated with 50 nM narciclasine generated a neurite area that was significantly reduced (Fig. 2B). As part of the standard operating procedure (SOP), acceptance criteria were defined that described the limit of variation acceptable within a plate for inclusion into data analysis. These were (i) a neurite area of at least 45,000 pixels per well (cell number and frames per well were constant) in the solvent control wells, (ii) a viability (viable cells = double positive for calcein and Hoechst) of > 90% in solvent control wells, (iii) a reduction of neurite area in cells treated with narciclasine (50 nM) by at least 25% relative to DMSO control and (iv) viability of the narciclasine-treated cells greater 90% relative to DMSO control. Analysis of these acceptance criteria across 36 test plates, run on 12 different days, showed a robust performance of the assay (Fig. 2C, D).

### 3.3 Workflow for screening and data analysis of the NeuriTox test

For the actual screening procedure, a defined workflow was established that contained several pre-determined decision points. All compounds were tested at 1:1000 dilutions of their stock concentration, and at subsequent 3-fold dilution levels. For most compounds, the stock was 20 mM, so that the test concentrations were 20 μM, 6.7 μM, 2.2 μM, 0.7 μM, 0.25 μM, 82 nM, 27 nM, 9 nM, 3 nM, and 1 nM. For 2,2’,4,4’,5,5’-hexabromodiphenyl-ether, chrysene, dibenz(a,h)anthracene, bis(tributyltin) oxide, benzo[g,h,i]perylene, and 2,3,7,8-tetrachlorodibenzo-p-dioxin lower stock concentrations were used.

Data, expressed relative to DMSO solvent control, were used for log-logistic 4-parameter curve fitting (11 data points for each of the two endpoints: neurite area (NA) and viability (V)). Subsequently, hit identification was conducted in a two-step process. Initially, compounds were classified as “active” if they affected NA or V significantly (one-way ANOVA and Dunnett’s post-hoc test) at any test concentration, or when they reduced NA or V by ≥ 20%. Otherwise, they were classified as “inactive compounds”. For active compounds, it was examined in a second step whether there was at least one concentration tested that affected NA significantly, but did not decrease V significantly. If this was the case, the compound was classified as a “hit compound”. Compounds that affected NA and V similarly at all concentrations, were classified as unspecific “cytotoxic compounds”.

**Fig. 3:**
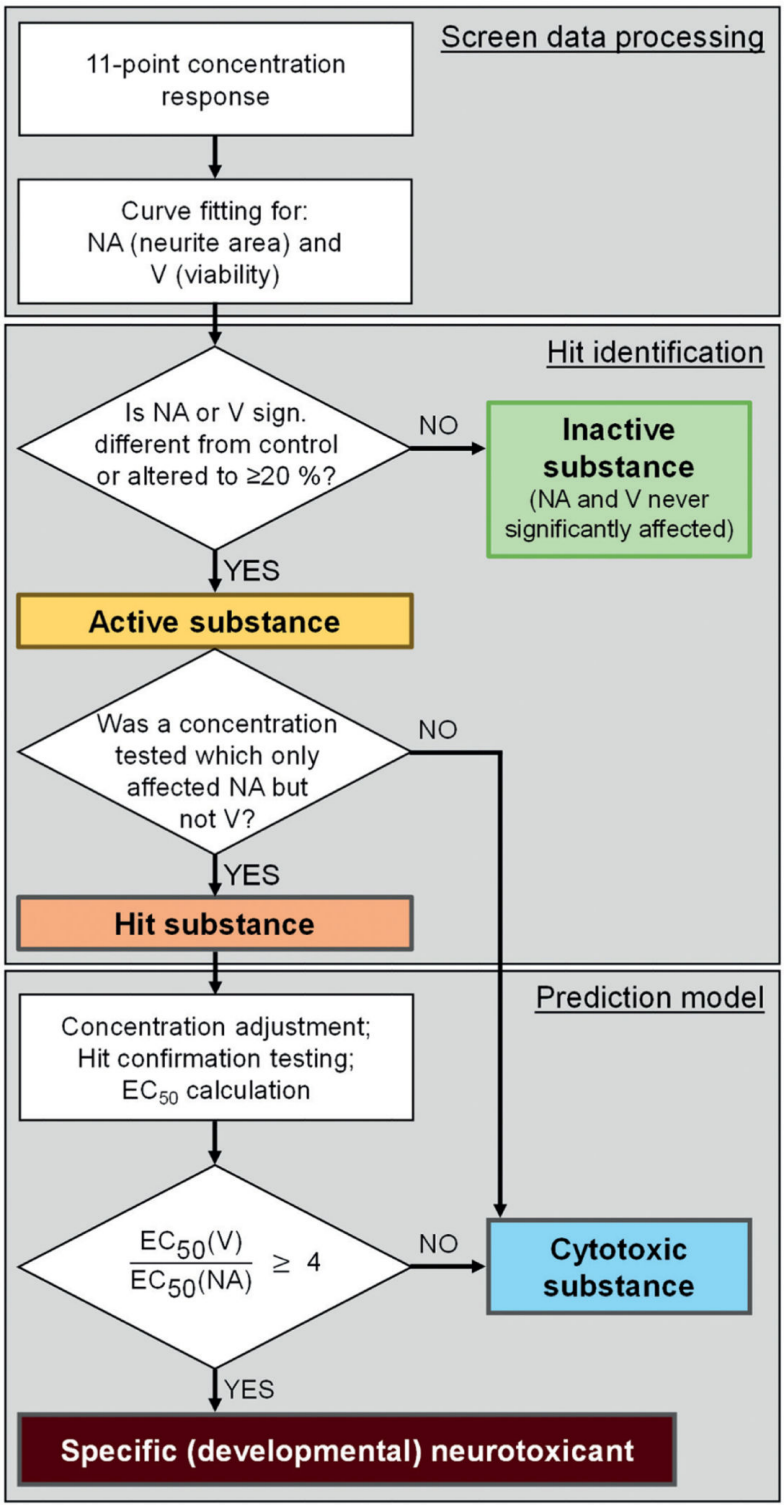
Workflow for screening and data analysis of the NeuriTox test During the screening, each NTP80 compound was tested at 10 concentrations (plus solvent control), starting at a maximum concentration of 20 μM and then in three-fold dilutions. Altogether, the screen was performed three times (independent biological replicates). Curve fitting was performed for neurite area (NA) and viability (V). If NA or V was neither significantly different from solvent control nor altered to more than 20%, the compound was classified as inactive. Otherwise the compound was considered to be active. If active compounds affected NA at more than one concentration that did not affect V, they were classified as “hits”. These were retested independently in an adjusted concentration range with subsequent curve fitting and EC_50_ calculation. If the EC_50_ ratio of V/NA was ≥ 4, the compound was classified as a “specific (developmental) neurotoxicant”. Otherwise it was classified as “cytotoxic”.

For the primary hits (= “hit compound”), hit confirmation testing was conducted. For that, the tested concentration range was adjusted to optimally span the estimated EC_10_, and three new independent experiments were performed. EC_50_ values were calculated according to the same procedure as for the screen data. Then, the EC_50_ ratio of V and NA, i.e., the offset of neurite outgrowth vs viability decrease, was calculated. These data were used as basis for the assay prediction model. If that ratio was ≥ 4, the compound was classified as a “specific (developmental) neurotoxicant”, while the compound was graded as a “cytotoxic compound” if the ratio was < 4 (Fig. 3).

### 3.4 Overview of NeuriTox screen results

**Fig. 4:**
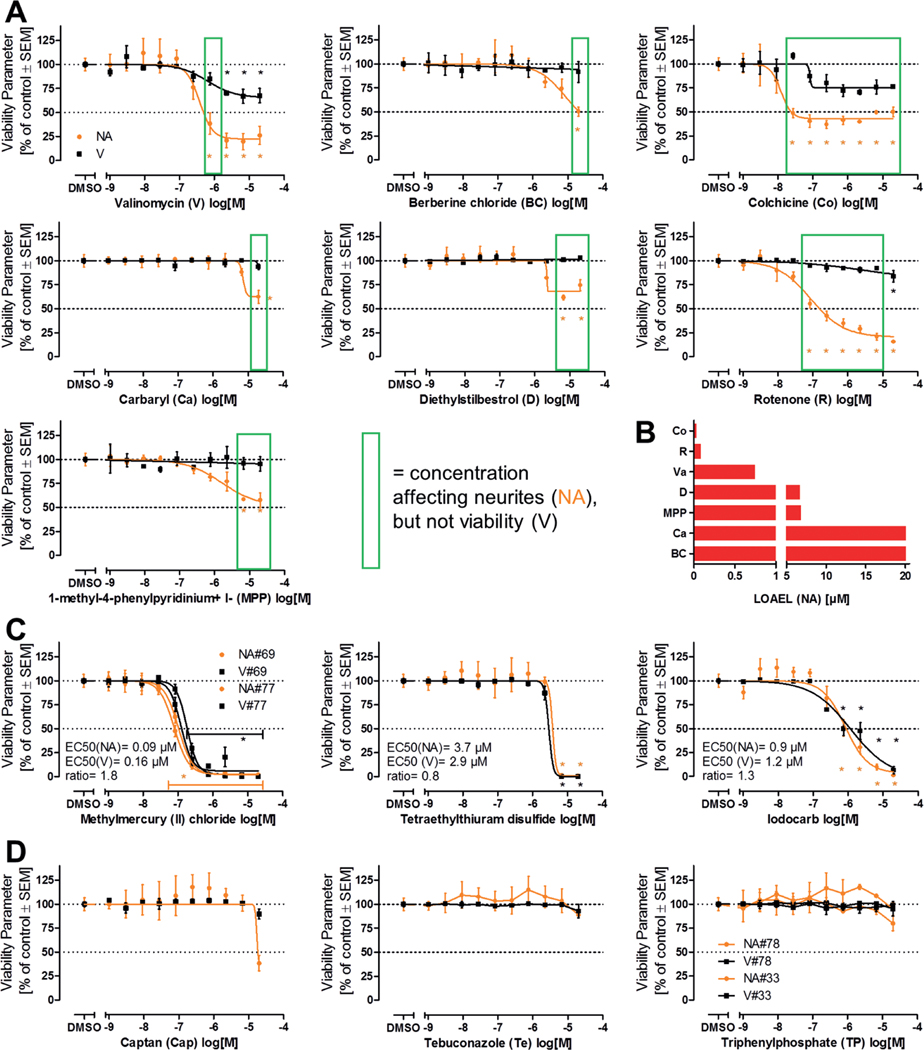
Overview of NeuriTox screen results A) LUHMES cells differentiated for two days were plated at a density of 100,000 cells/cm^2^ (ca. 30,000 cells/well) into 96-well plates, treated one hour later and analyzed after 24 h. Neurite area (NA, orange) and viability (V, black) were determined by high content imaging. Concentration-response curves are given for compounds that were classified as hits. Green boxes outline concentrations which only affected NA but not V. B) Comparison of lowest observed adverse effect levels (LOAEL, lowest experimentally tested concentration that resulted in a change that was statistically significant from control) for NA of screen hits. C) Examples for concentration response curves for cytotoxic compounds without specific neurite effects. EC_50_ concentrations are indicated for NA and V as well as their ratio. D) Examples for three compounds which gave ambiguous responses in the screen (apparent drop of NA at 20 μM vs control or vs low (1 nM) concentration). All data are means ± SEM from three biological replicates, dotted lines are drawn at 100% and 50%. #: compound position on the library plate if compound was present twice. *, p < 0.05 by one-way ANOVA followed by Dunnett’s post-hoc test.

After testing of all “80 compounds” in three independent experiments in the NeuriTox test, concentration-response graphs were produced for subsequent data analysis. Seven compounds (valinomycin, berberine chloride, colchicine, carbaryl, diethylstilbestrol, rotenone and MPP^+^) caused a significant decrease in neurite area at concentrations that did not affect viability (Fig. 4A). Therefore, these compounds were classified as “active hit compounds”. The lowest tested concentration that evoked an adverse effect (i.e., statistically significant reduction in neurite area compared to control) ranged between 27 nM (colchicine) and 20 μM (carbaryl and berberine chloride) (Fig. 4B).

Most cytotoxic compounds affected the neurite area and cell viability to about the same extent at any tested concentration (Fig. 4C, Fig. S5^1^). As methylmercury (II) chloride was present as duplicate on the master plate, it was tested twice (as compound #69 and #77). The resulting curves overlapped to a large extent, indicating assay robustness and reproducibility. EC_50_ values were calculated for clear cytotoxicants directly from the screen results.

Unclear screen results were obtained only for some compounds (Fig. 4D). In these cases, the available data were not considered sufficient for classification of the respective chemical (e.g., as a neurotoxicant). Therefore, three compounds were re-tested. Captan proved to be a cytotoxicant with relatively low potency, while tebuconazole and triphenylphosphate (the latter was present twice in the library) were clearly non-toxic at concentrations up to 20 μM (Fig. 4D, Fig. S6^1^), resulting in a toxicity EC_50_ of 238 μM for tebuconazole and > 200 μM for triphenylphosphate, when an extended concentration range was examined.

### 3.5 Hit confirmation testing and hit definition in the NeuriTox test

**Fig. 5:**
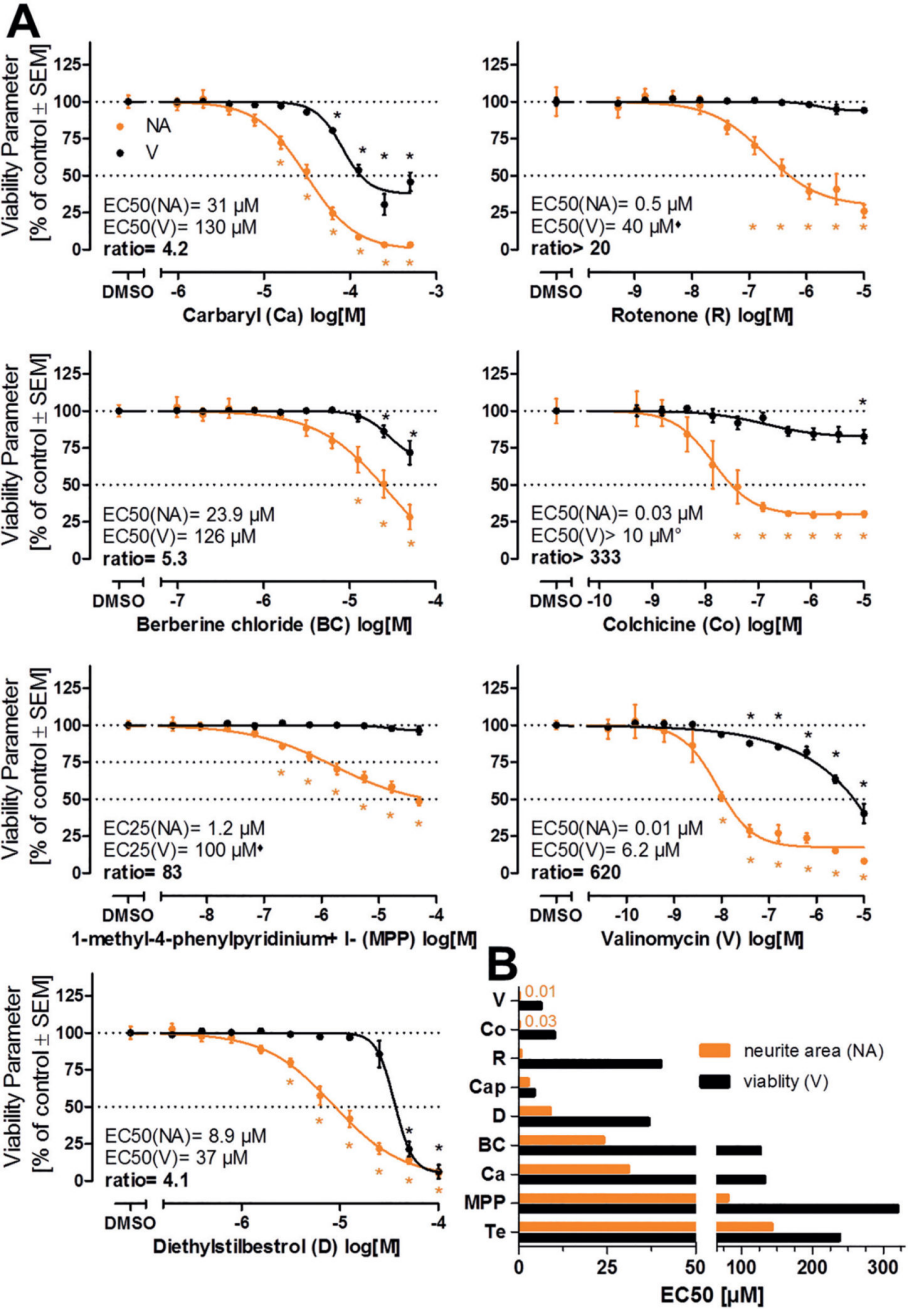
Results of NeuriTox hit confirmation testing A) Compounds that were classified as hits after the first round of screening were re-ordered independently and re-tested for their effect on neurite outgrowth inhibition (NA, orange) and viability (V, black) in an adjusted concentration range (otherwise same experimental setup as for the screening). EC values and their ratios were calculated from four-parameter log-logistic fit functions. The curves for the compounds that were classified as specific (developmental) neurotoxicants are displayed. B) Comparison of the EC_50_ values for neurite area and viability of the compounds that went through hit confirmation testing. If the EC_50_ ratio V/NA was ≥ 4, the compound was classified as a specific (developmental) neurotoxicant (not for captan (Cap) and tebuconazole (Te)). Detailed explanations for calculations and rule sets applied in case of curves not hitting the 50% level (°, ♦) are given in the methods description. All data are means ± SEM from three biological replicates, dotted lines are drawn at 100% and 50%. *: p < 0.05, by one-way ANOVA followed by Dunnett’s post-hoc test.

For hit confirmation testing, the compounds were re-purchased, and new stocks were prepared. Re-testing was performed using the same method, but within an optimized concentration range to facilitate EC_50_ calculation. The limits to this were compound solubility and a maximum DMSO concentration of 0.33% (v/v) in the NeuriTox test. All compounds that were identified as “active hit compound” in the screen were confirmed (Fig. 5A). The EC_50_ ratio of the compounds classified as “active hit compounds” ranged between 4.1 (diethylstilbestrol) and 620 (valinomycin) in the hit confirmation. Therefore, these seven compounds were classified as specific (developmental) neurotoxicants (Fig. 5B). Concerning the potency of the hit compounds, the EC_50_ values for reduction of neurite area ranged from 10 nM (valinomycin) up to 80 μM (MPP^+^). Even lower potencies were seen for some compounds, e.g., for tebuconazole (142 μM), but the latter compound was not a specific neurotoxicant according to our prediction model.

We were interested to what extent the hit definition depended on the prediction model. Here, we used the well-established prediction model of the NeuriTox test (based on EC_50_ values) as the default method (Krug et al., 2013; Stiegler et al., 2011). It was developed specifically for this assay, and it differs (i) from that used for other assays of neurite growth (Harrill et al., 2013; Harrill and Mundy, 2011; Flaskos et al., 2011; Frimat et al., 2010; Howard et al., 2005; Yang et al., 2014), (ii) from that of other assays that screened the same library (Ryan et al., 2016; Sirenko et al., 2017), and (iii) even from other assays developed in our own laboratory (Nyffeler et al., 2017a). If multiple assays are to be compared, as in the Tox21 program (Judson et al., 2014, 2016, 2015; Tice et al., 2013) or in screening the NTP80 collection, it may be advantageous to use a more generalized algorithm for hit definition. One such approach, taken by the NTP, uses the concept of benchmark concentrations (BMC). The underlying idea is that hits are defined by their distance from the background noise of a given assay. In more mathematical terms, the following steps are taken: (i) the standard deviation of negative controls is determined (= background noise level, BN); (ii) this information is used to define a benchmark response (BMR), which follows the same rule for each assay (e.g., BMR = 3 x BN); (iii) a concentration-response curve is fitted through the test compound data; (iv) the intersection of this curve with the BMR level is determined; (v) the concentration of the compound corresponding to this intersection-point is determined as the BMC. This procedure was applied here both for the viability and for the neurite area data obtained in the NeuriTox screen (Fig. S4A^1^).

The identification of active compounds obtained by this BMC method was largely similar to the prediction model of the NeuriTox assay (Fig. S4B^1^). Differences were only observed for the classification of “borderline compounds” into cytotoxic or specific developmental neurotoxicants. In such cases, one or the other approach may be more sensitive or specific (depending on variations and type of uncertainty of the test data). It was obvious that also the setting of the specificity-thresholds affected hit identification. For instance, if specificity of a compound was classified by the BMC ratio (BMC_V_/BMC_NA_), and the threshold was set at 3.16 (Ryan et al., 2016), valinomycin and carbaryl were classified as cytotoxic. If that threshold was changed to 2, the BMC method classified the same compounds as specific DNT-compounds, in agreement with the EC_50_ method (Fig. S4B^1^).

Three compounds, bisphenol A (BPA), 2,2’,4,4’-tetrabromodiphenylether (TBDE), and 2,2’,4,4’,5’-pentabromodiphenylether (PBDE) showed larger than normal data variation in the screen. According to the NeuriTox data analysis workflow, all three were initially classified as “active”. These compounds were also active according to the BMC method. However, re-testing of BPA showed that it actually has no effect up to 100 μM (data not shown), while the others retained the classification as “unspecific cytotoxicants”.

In summary, this comparison of entirely different hit definition approaches showed that they mostly lead to similar results. This suggests that the test method is robust. Moreover, it shows that both approaches may be useful, depending on the intended use: the BMC method may be more sensitive (fewer false negatives), and it is less dependent on the part of the curve that reflects high toxicant concentrations. On the other hand, it depends greatly on the quality of the data in the low toxicity range.

### 3.6 Chemical characterization of specific hit compounds (= specific (developmental) neurotoxicants)

To elucidate whether the NeuriTox test has a bias to detect compounds with certain physicochemical properties, these were investigated for the set of tested compounds. Compounds that were identified as specific neurite outgrowth inhibitors in the NeuriTox test were analyzed regarding their hydrophobicity and molecular weight (Fig. S2A^1^). While there was no bias for a certain molecular weight detectable, all identified specific neurite outgrowth inhibitors were located in a medium hydrophobicity range (logP values 0–5). A more generalized approach, using hundreds of chemical descriptors (GRIND2 physicochemical descriptors), showed that the specific neurite outgrowth inhibitors were evenly distributed within the physicochemical properties of the NTP80 collection compounds, and even within the chemical space of the large libraries Tox21 and DrugBank (Fig. 1C and Fig. S2B^1^). These data suggest that the NeuriTox test has no obvious classification bias with respect to physicochemical properties.

### 3.7 NeuriTox hits in light of Tox21 data on these compounds

**Fig. 6:**
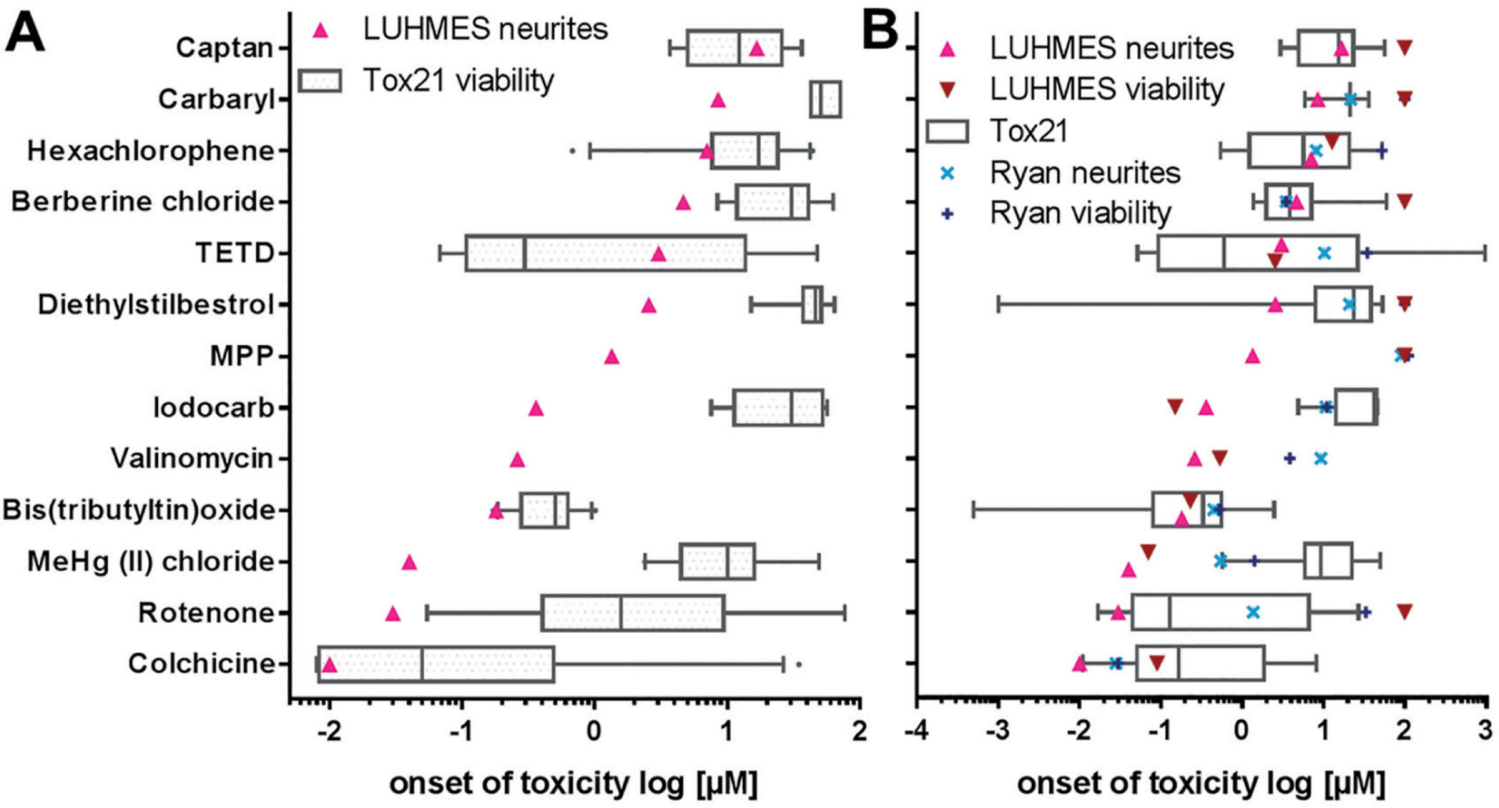
Comparison of NeuriTox data with Tox21 data sets A) Benchmark concentrations (BMC) were calculated as a measure for “onset of toxicity” for all active compounds across different assays. The BMC values for neurite outgrowth inhibition of LUHMES cells were in the range of the EC_25_ values. They were visualized together with BMC viability data of the Tox21 data set (n = 168 viability endpoints in total, 7–28 per compound). B) Comparison of the BMC for neurite outgrowth inhibition of active NeuriTox assay compounds with BMC values for specific functional endpoints (e.g., receptor activation, stress response signaling) assessed in the Tox21 screening (n = 123 specific endpoints in total, 8–16 per compound), excluding viability data, and versus a previously published neurite outgrowth test method (Ryan et al., 2016). If no BMC could be calculated for NeuriTox or the test method reported by Ryan, it was set to 100 μM for visualization reasons. Boxes display the 25^th^ and 75^th^ percentiles as well as medians. The whiskers span from the 10^th^ to 90^th^ percentiles, data points beyond these limits are displayed as individual points.

For all active compounds identified from the NTP80 collection by the EC_50_ and BMC method, available data were extracted from the Tox21 database. In order to compare data from different assays, the BMC value for the most sensitive measured endpoint was used. On this basis, the impairment of LUHMES neurite outgrowth was compared with all viability data in the Tox21 library (boxes and whiskers, n = 168 viability endpoints in total, 7–28 per compound) (Fig. 6A). For 11 of the 13 compared compounds, inhibition of LUHMES neurite outgrowth was more sensitive than the median response of the Tox21 assays; for 10 of the 13 compounds, LUHMES cells were even more sensitive than the 25^th^ percentile fraction of the Tox21 viability results. No Tox21 results were available for valinomycin and MPP^+^.

Furthermore, LUHMES neurite outgrowth was compared against functional endpoints (e.g., receptor activation or stress response signaling (n = 123 specific endpoints in total, 8–16 per compound) measured in the Tox21 set up, excluding viability measurements. Data from a recently published neurite outgrowth test method were included in this comparison (Ryan neurites, Ryan viability, Fig. 6B) (Ryan et al., 2016).

For 9 of the 13 compounds, LUHMES neurite outgrowth was a more sensitive endpoint than the median of all functional Tox21 data. For all compounds, except for berberine chloride, the NeuriTox test was more sensitive than the alternative neurite outgrowth test method used by Ryan. The parkinsonian toxicant MPP^+^ was only detected in the NeuriTox test. This is consistent with its known mode of action, which requires the dopamine transporter. The latter is expressed in LUHMES cells (Lotharius et al., 2002; Schildknecht et al., 2009), but not in the mixed neuronal cultures used by Ryan et al. (2016).

### 3.8 Re-testing of the NTP collection in the PeriTox test

**Fig. 7:**
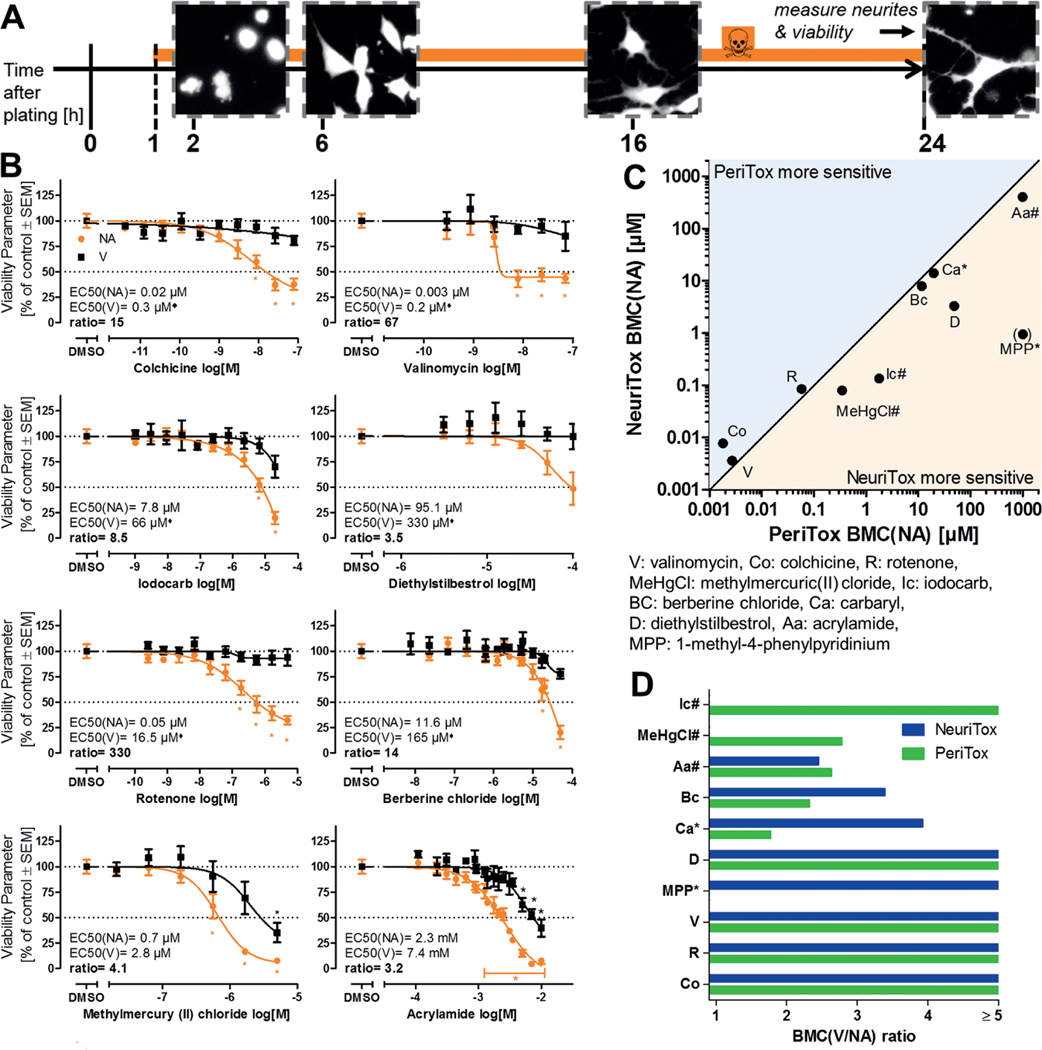
Comparison of the PeriTox test results with NeuriTox test hits A) Exposure scheme for PeriTox test. Immature human dorsal root ganglia neurons were differentiated from pluripotent stem cells and frozen. For testing, cells were thawed and plated into 96-well plates (left edge of scheme). Treatment was initiated at one hour after plating. After 24 h, nuclei were stained with H-33342 and the cytoplasm was stained with calcein-AM. Then cells were imaged and an algorithm was applied that identified the neurite area and viability. Representative images of the calcein stain are shown for the different times after seeding. Image width is 175 μm. B) The NTP80 collection of compounds was screened in the PeriTox assay. Neurite area (NA, orange) and viability (V, black) were determined in at least six concentrations to identify hits. Compounds identified as hits in the PeriTox screen were re-tested and hit confirmation data are displayed. EC values and their ratios were calculated from four-parameter log-logistic fit functions. Compounds with an EC_50_ ratio (V/NA) ≥ 3 were classified as specific hits in the PeriTox test. The rule set used for ratio calculations is specified in the methods section. All data are means ± SEM from three biological replicates, dotted lines are drawn at 100% and 50%. *: p < 0.05, by one-way ANOVA followed by Dunnett’s post-hoc test. C) To visualize differences between central nervous (NeuriTox) and peripheral (PeriTox) neuropathy hazard, BMC values for neurite outgrowth inhibition of PeriTox were plotted against NeuriTox test BMC data. Compounds marked with # were only specific in PeriTox, whereas compounds marked with * were only specific in NeuriTox; compounds without an extra mark where specific hits in both tests. The light-blue area marks where the PeriTox test was more sensitive, whereas the light-yellow area indicates where the NeuriTox test was more sensitive. For visualization reasons, MPP^+^ was assigned an arbitrary BMC(NA) of 1000 μM for the PeriTox test, although it was not active at all. D) Comparison of the BMC(V/NA) ratios of the compounds that were classified as specific hits in either the NeuriTox or PeriTox tests. Blue bars represent the ratios for NeuriTox, green bars for PeriTox. For visualization reasons, ratios > 5 were cut. Compounds marked with # were only specific in PeriTox, whereas compounds marked with * were only specific in NeuriTox.

We were interested in how far hits in the NeuriTox screen (hazard of compounds to central nervous system neurons) would overlap with the activity of compounds of the NTP80 collection in a recently established (Hoelting et al., 2016) test of peripheral neurotoxicity (PeriTox test). This method uses human immature dorsal root ganglia neurons (iDRG) that are produced from pluripotent stem cells that are still in a phase of neurite growth. Like in the NeuriTox assay, exposure to toxicants in this test is for 24 h, and readouts for viability (V) and neurite area (NA) are also conducted in a similar way (Fig. 7A).

Three independent screen runs were performed, and eight compounds were identified as “active hit compounds” according to the evaluation algorithm specified above for the NeuriTox test. These compounds (berberine chloride, carbaryl, colchicine, diethylstilbestrol, rotenone, valinomycin, iodocarb, and methylmercury chloride) underwent subsequent hit confirmation testing. Seven of the eight compounds were confirmed as specific hits according to the published prediction model (EC_50_ ratio viability/neurite area ≥ 3; Hoelting et al., 2016). Carbaryl failed this verification step (Fig. 7B, Fig. S7^1^). After the screen, we included acrylamide in the group of hits. This is known from a former publication (Hoelting et al., 2016) to be a specific and active compound in the PeriTox assay, but at concentrations ≥ 20 μM (Fig. 7B). As further post-testing step, valproic acid (VPA) was classified as cytotoxic. This was done on the basis of previously obtained data in a much higher concentration range than used in the screen (Fig. S7^1^). For carbaryl and VPA, the ratio of the EC_50_ values for NA and V was > 2 but < 3. We investigated alternative prediction models (BMCs, EC_30_, EC_25_ and EC_20_ ratios, Fig. S7^1^) to explore whether they would indicate a specific effect of the toxicants. However, a ratio > 3 was reached by neither approach. Thus, the default prediction model appears to yield a robust definition of hits and non-hits.

For the PeriTox hits, the potency spanned a range from 20 nM (EC_50_(NA)) for valinomycin to 2.3 mM (EC_50_(NA)) for acrylamide. The offset between adverse effects on NA and V (EC_50_ ratios) ranged from 3.2 (acrylamide) up to 330 (rotenone). These ratios were useful ranking measures within the PeriTox assay. However, they are based on various assumptions (e.g., curve shape and steepness) and they were therefore considered problematic for comparisons with other assays (e.g., the NeuriTox test). In order to directly compare the effects of the same set of compounds on different tests (NeuriTox vs PeriTox), the BMC(NA) values (referring more to the onset of toxicity) were plotted for compounds which were identified as specific compounds in at least one of the tests (Fig. 7C). This approach allowed the comparison of the hazard to the central nervous system (NeuriTox) vs the peripheral nervous system (PeriTox). The NeuriTox assay showed a tendency to be affected at lower compound concentrations when the compound was a hit. The PeriTox had a higher hit-rate (detection of acrylamide, iodocarb and methylmercury chloride). The PeriTox detected acrylamide, a well-known peripheral neurotoxicant (Cavanagh, 2000; Spencer and Schaumburg, 1975), whereas the NeuriTox assay identified MPP^+^ as a hit, well in accordance with the known central nervous toxicity (Schildknecht et al., 2017) of this compound (Fig. 7C).

For comparison of the specificity (V/NA ratios) of the tests, the default prediction models have disadvantages (rules dealing, for example, with viability curves that did not drop to a 50% level). Thus, BMC values were used. This comparison shows that there are indeed some drastic differences (e.g., for MPP^+^). It also demonstrates that some differences in the identification of specific hits are not very robust. For instance, acrylamide, re-tested at high concentration in the PeriTox and NeuriTox assays, was a specific PeriTox hit according to the individual test prediction models. Comparison of BMC ratios suggests however, that the differences between the tests are minor. In such a borderline situation, a compound may end up by chance on either side of the hit threshold, and for some purposes, it would be useful to introduce a third category (besides hits and non-hits) of “borderline compounds” (Fig. 7D).

The comparison also clearly shows the advantage of using two complementary assays for the same type of endpoint if sensitivity of compound identification (e.g., for further testing) is a major issue. The combination of both tests had a higher sensitivity for detection of potentially hazardous compounds.

### 3.9 Comparison of data from neurite toxicity assays with other published DNT tests

**Fig. 8:**
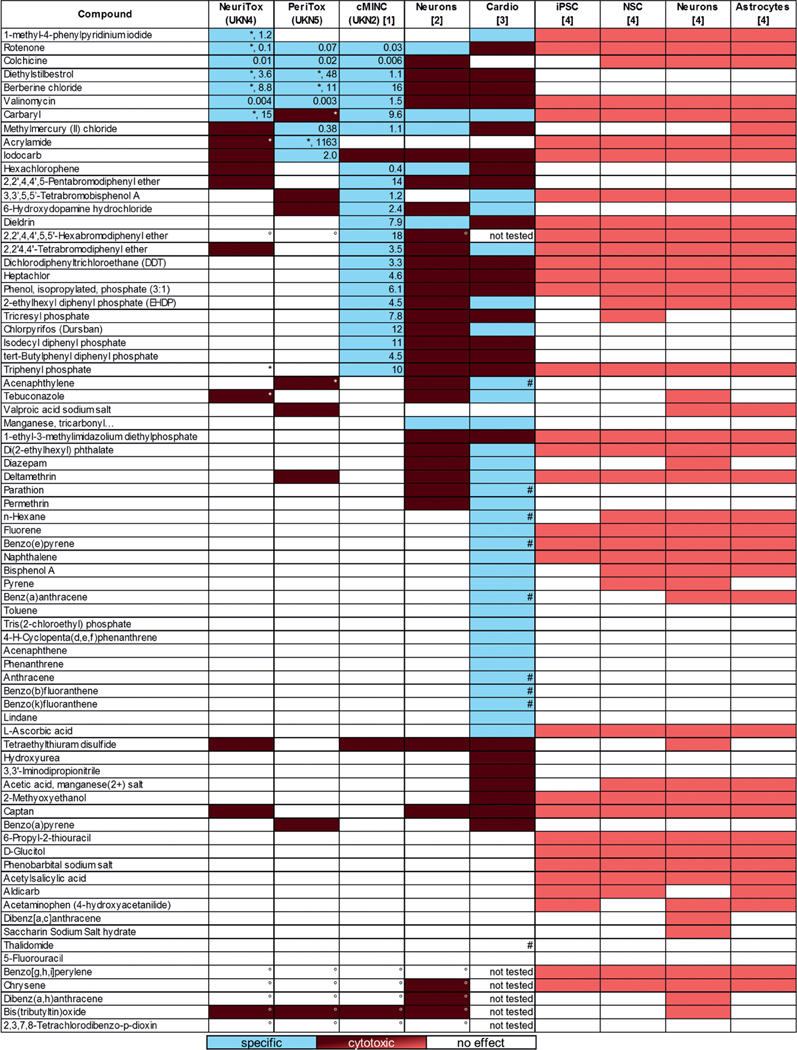
Cross comparison of test data for the NTP80 collection NeuriTox (= UKN4) and PeriTox (= UKN5) data obtained here are shown in the context of published data from other test runs on the NTP80 collection. The effect of the compounds on the different tests is indicated as specific effect on cell function (blue), cytotoxic effect (red) or no effect (white); light red coloring indicates that the used assay did not discriminate between specific effects and cytotoxicity (Pei et al., 2016). For the specific hits of the NeuriTox, PeriTox and cMINC tests (Nyffeler et al., 2017a,b), the EC_25_ for the most sensitive endpoint is given in μM. For the NeuriTox test, specific hits were defined by an EC_50_(V/NA) ratio of ≥ 4, for the PeriTox test the ratio had to be ≥ 3. For the cMINC test, compounds inhibiting migration to ≥ 25% without affecting viability by more than 10% were considered specific. For the alternative neurite outgrowth model (Ryan et al., 2016), specificity was defined as ratio between BMC concentrations for viability and neurite area ≥ 3.16 and the confirmation of this classification in a retesting. In the cardiotoxicity test (Sirenko et al., 2017), compounds were defined as specific if they i) affected cardio-physiologic parameters after 30 min treatment at a three-fold lower concentration than viability and ii) if they had no effect on viability after 24 h. If not stated otherwise, NeuriTox, PeriTox and cMINC were performed with 20 μM as highest concentrations (with a DMSO concentration of 0.1%). Other assays were performed at up to 100 μM (with up to 0.5% DMSO in the test). An asterisk (*) indicates that the compound was tested at higher than standard concentrations, ° indicates that a compound was tested at lower than standard concentrations, # indicates that the calcein signal was impaired, but the authors did not conclude cytotoxicity from that.

Hazardous effects of the NTP80 collection have so far been described in four publications, which span a pure cytotoxicity assessment of cells in varying neural differentiation states (Pei et al., 2016), an alternative neurite outgrowth model (Ryan et al., 2016), and highly function-based studies focusing on the migration of neural crest cells (Nyffeler et al., 2017a) or adverse effects on cardiomyocyte function (Sirenko et al., 2017). These data were synoptically compared to the results of our study (Fig. 8).

In the first published screen (Pei et al., 2016), cytotoxicity was assessed after exposure to the NTP80 collection compounds at two different concentrations (10 and 100 μM) for 72 h. In this study, many compounds appeared cytotoxic to neural cells, but hit confirmation was not performed. On the other hand, the cardiotoxicity screen (Sirenko et al., 2017) addressed a broad set of endpoints and more than half of the 69 tested compounds affected cardiomyocytes in some way. A prediction model still also needs to be developed for that test method. For our comparison, we ranked only those compounds as potentially cardiotoxic, which i) affected cardio-physiologic parameters after 30 min treatment at a three-fold lower concentration than viability and ii) if they had no effect on viability after 24 h. For the Ryan et al. (2016) neurite outgrowth model, we adopted the classification suggested by the authors: a specific neurotoxin had a BMC for neurite outgrowth that was at least 3.16-fold (= one half-log dilution step) lower than for general cytotoxicity. For the neural crest migration (cMINC) (Nyffeler et al., 2017a) as well as for the NeuriTox and PeriTox tests, the published prediction models were used (Hoelting et al., 2016; Krug et al., 2013).

Limiting the comparison to compounds selectively active for neuro or cardio effects, the cMINC test and the cardiotoxicity assay classified the highest number of compounds (23 and 32, respectively). Further analysis of the specific compounds showed that none of the tested compounds evoked a specific response in all assays. However, seven of the 69 compounds (rotenone, diethylstilbestrol, berberine chloride, valinomycin, carbaryl, methylmercury(II)chloride, and iodocarb) were active (not necessarily specific) in all test methods when full concentration responses were considered.

Comparing which compounds were classified as specific between neural (NeuriTox, PeriTox, cMINC) cell based tests and the cardiotoxicity test method showed that many compounds that were specific in the neuronal system were generally cytotoxic in the cardiomyocyte-based test method (e.g., rotenone, diethylstilbestrol, berberine chloride, valinomycin), whereas compounds that were specific in the cardiotoxicity test method were inactive or generally cytotoxic in the neuronal-based tests (e.g., carbaryl, hydroxydopamine). From this initial comparison of tests, the PeriTox and NeuriTox tests appear to have a largely overlapping specificity range. Moreover, most hits of the neurite assays are also identified by the cMINC test. However, the latter test identifies a large group of additional compounds. The cardiotoxicity test method seems to be largely complementary.

## 4 Conclusion and outlook

Our comparative compilation of screen data shows where gaps remain to be filled in data generation and interpretation. For instance, strong developmental toxicants, such as thalidomide and 5-fluorouracil, were not detected by any of the published screens. This pinpoints the need for supplementing the test battery with other complementary tests.

Our comparison also revealed some technical issues that need to be addressed:

- The definition of non-actives is difficult, especially if the highest tested concentration differs between screens.
- The use of nominal concentrations for comparisons poses problems, as the compound concentration that impacts the cell directly is influenced by its physicochemical parameters (e.g., extent of adhesion to plastic and serum binding), and the properties of the test system (cell density, medium type). The free soluble concentration in the culture medium or the intracellular concentration would provide more comparable measures.
- Different concentrations of solvent (e.g., 0.1% (v/v) DMSO (= 14 mM) or 0.5% (v/v) DMSO (= 70 mM)) can affect screen results.
- Fixed concentration range screens that limit the highest possible concentration prevent testing of low potency but highly abundant compounds at relevant exposure concentrations. Examples here were VPA and acrylamide, where clinical and accidental exposure can be higher than the highest tested concentration used in our screen. The issue of test concentrations is also important in another context: how do the concentrations at which hits are observed relate to relevant *in vivo* concentrations? This point was neglected here, since the screen is designed to create alerts, and the follow-up evaluation would then prioritize them, e.g., taking various exposure scenarios and related estimates of human brain, plasma or fetal concentrations into account.
- The different false-positive rates of screens are important for comparison of screen hits or for subsequent toxicological evaluations (e.g., for QSAR or read-across approaches). In order to obtain a good sensitivity (low number of false-negatives), hit definitions of screens are set in a way to allow many false-positives. For instance, if the significance level is set to 0.1, then a screen of 80 compounds will result in 8 false-positives. This number can subsequently be drastically reduced by secondary re-testing of hits.
- One of the most pertinent issues of hit definition is the test prediction model. Most screens, including NeuriTox and PeriTox, use a binary model (hit/non-hit). In such cases, threshold setting requires a large learning and training set of negative, unspecific and positive control compounds (Crofton et al., 2011; Leist et al., 2010; Schmidt et al., 2017). For the NeuriTox and PeriTox tests, prediction models have been established based on the evaluation of the EC_50_ ratio of viability and neurite area (Hoelting et al., 2016; Krug et al., 2013; Stiegler et al., 2011). These prediction models are designed in a way that compounds that affect neurites more potently than viability (EC_50_(V/NA) ratio > 4 for NeuriTox and EC_50_(V/NA) ratio > 3 for PeriTox) are considered specific neurotoxicants. It is important to note that the prediction model only makes a statement on positives (= neurotoxicants). The model does by no means imply that compounds with a low EC_50_ ratio (= non-hits) are non-toxicants. This potential fallacy must be strictly avoided. For instance, strong cytotoxicity under the given *in vitro* test conditions may mask a potential specific *in vivo* neurotoxic effect.
- Furthermore, it has to be considered which curve-fitting approach and constraints were applied to yield summary data from the curve-fit to enter them into the prediction model. For instance, EC_50_ values are relatively robust against baseline fluctuations, but they depend strongly on the shape of the concentration response curve (shallow vs steep) and on the lower part (higher toxicity range) of the curve. In contrast, BMC values better define the actual onset of toxicity, but they depend strongly on the low-concentration data and baseline fluctuations. If the focus on data analysis is strong sensitivity (low false-negative rate) or comparison across many different models, the BMC is a very useful method.
- An issue that may also need to be revisited in the future is the classification of so-called “borderline compounds”, where the EC_50_ ratio is close to the specificity threshold. Following the classical binary prediction model of, e.g., the NeuriTox test, a compound with an EC_50_ ratio of 3.9 is classified as “cytotoxic”, whereas a compound with a ratio of 4.1 is a “neurotoxicant”. This sharp distinction contrasts with the statistical variation of data, e.g., in different screen runs. Therefore, it might be helpful to introduce a third category of “borderline compounds”, which comprises the range around the threshold (Leontaridou et al., 2017). Alternatively, a probability-based prediction model could be developed which is not based on distinct hazard classes (i.e., “non-specific”, “borderline”, “specific”) (Leontaridou et al., 2017), but which identifies the compound’s hazard potential (Paparella et al., 2017, 2013; Leist et al., 2014; Basketter et al., 2012; Jaworska and Hoffmann, 2010; Hartung et al., 2013; Judson et al., 2015). An example for such an approach is given in Fig. S8^1^, but considerable further work is required to refine this approach.
- Further issues are acceptance criteria for test data and resultant curve shapes. Here, we used “inspection by the human eye” to ensure some plausibility (e.g., monotonic curve shapes). This procedure may introduce bias, and it is difficult to apply to large screens. An example from the NeuriTox screen is the concentration response curve for tebuconazole (Fig. 4D). This compound never affected viability or neurite area more than 20% and it would therefore be classified as a non-hit. However, visual inspection showed a non-monotonic concentration-response curve. It was therefore re-tested and was indeed identified as an active cytotoxic compound (at high concentrations) (Fig. S6^1^).
- Of toxicological concern are false negatives due to biological differences of the screen system vs the *in vivo* situation. A typical example here is the non-toxicity of hexane, a known neurotoxicant. *In vivo*, hexane is activated by P450 enzymes to hexanedione and this metabolite subsequently causes neurotoxicity. The lack of a metabolite activation system prevents the detection of such toxicants. Similarly, the *in vitro* system may lack important toxicant targets or the readout used can be independent of a certain target activity. An example here is acetylcholinesterase (AChE), which does not play a role for the assay readout, and thus, typically neurotoxic AChE inhibitors are not detected.

In order to transit from screen hits to more definite toxicological statements, it is usually necessary to test whether a similar adverse effect can be confirmed with another test method (= secondary hit follow-up) (Nyffeler et al., 2017a). Here, the PeriTox test was used to gain further information on potential neurotoxic hazard. It needs to be noted that the NeuriTox and PeriTox assays do not test for identical toxicities but are complementary to a certain extent. While the NeuriTox test was designed to identify toxicants that disrupt neurites of the central nervous system neurons, the PeriTox test was set up to detect peripheral neurotoxicants. This complementarity is exemplified by the fact that the parkinsonian toxicant MPP^+^ is only picked up by the NeuriTox test, while, e.g., acrylamide was only identified as specific hit by the PeriTox test. However, since both tests are based on the impairment of neuronal structures, a certain degree of convergence is expected as well. Both tests identified the same five compounds (out of 7 or 8 specific hit compounds in the NeuriTox and PeriTox test, respectively) as specifically neurotoxic.

Knowing for which hazard assessment scenarios these tests can be applied, rationally structured test batteries can be built in an efficient (minimal overlap of tests) and sufficient (broad coverage of biological endpoints) manner.

## Supplementary Material

sup 1

sup 2

## Footnotes

1 doi:10.14573/altex.1712182s1

2
http://www.rdkit.org

3 https://github.com/flatkinson/standardiser (accessed 21.11.2016)

4
http://www.moldiscovery.com/software/pentacle

5 https://www.epa.gov/chemical-research/toxicity-forecaster-toxcasttm-data (retrieved on 29.07.2016; data released 19.10.2015)

6 doi:10.14573/altex.1712182s2

7 https://ntp.niehs.nih.gov/sandbox/tox21-activity-browser/ (accessed 28.06.2016)

## Abbreviations:

BMC: benchmark concentration
BN: background noise level
BPA: bisphenol A
BR: benchmark response
calcein-AM: calcein acetoxymethyl ester
cAMP: N6,2′-O-dibutyryladenosine 3′,5′-cyclic monophosphate
cMINC: neural crest cell migration test
DM: differentiation medium
DMSO: dimethyl sulfoxide
DNT: developmental neurotoxicity
EC: effective concentration
FGF: fibroblast growth factor
GDNF: glial derived neurotrophic factor
log: logarithm of the partition-coefficient
LUHMES: Lund human mesencephalic
MW: molecular weight
NA: neurite area
NT: neurotoxicity
NTP: National Toxicology Program of the USA
OECD: Organisation for Economic Co-operation and Development
PAH: polycyclic aromatic hydrocarbons
PCA: principal component analysis
PDM: PeriTox differentiation medium
PLO: poly-L-ornithine
PM: proliferation medium
SEM: standard error of the mean
UKN2: cMINC
UKN4: NeuriTox
UKN5: PeriTox
V: viability
VPA: valproic acid
