# Supplementary material for: A High-Throughput Approach to Identify Specific Neurotoxicants / Developmental Toxicants in Human Neuronal Cell Function Assays
Source: ALTEX. Author manuscript; Available in PMC 2023 Jun 14. (PMC10266261; doi:10.14573/altex.1712182)
Supplement: sup 1 [file NIHMS1893850-supplement-sup_1.pdf]

Delp et al.:

A High-Throughput Approach to Identify Specific Neurotoxicants / Developmental Toxicants in Human Neuronal Cell Function Assays

Supplementary Data

cmpd #	class	Compound	CAS#	logP	MW [g/mol]	NeuriTox (UKN4)	PeriTox (UKN5)	cMINC (UKN2) [1]	Neurons [2]	Cardio [3]	IPSC [4]	NSC [4]	Neurons [4]	Astrocytes [4]
5	drug/drug-like	1-methyl-4-phenylpyridinium iodide	36913-39-0	2.75	155.20	*, 1.2								
44	pesticide	Rotenone	83-79-4	3.70	394.42	*, 0.1	0.07	0.03						
35	drug/drug-like	Colchicine	64-86-8	2.87	399.44	0.01	0.02	0.006						
63	drug/drug-like	Diethylstilbestrol	56-53-1	4.83	268.36	*, 3.6	*, 48	1.1						
19	drug/drug-like	Berberine chloride	633-65-8	3.10	336.37	*, 8.8	*, 11	16						
2	drug/drug-like	Valinomycin	2001-95-8	2.34	1111.34	0.004	0.003	1.5						
41	pesticide	Carbaryl	63-25-2	2.56	201.23	*, 15	*	9.6						
68	env. t./heavy metal	Methylmercury (II) chloride	115-09-3		251.08		0.38	1.1						
33	industrial chemical	Acrylamide	79-06-1	-0.34	71.08	*	*, 1163							
42	pesticide	Iodocarb	55406-53-6	1.91	281.09		2.0							
29	pesticide	Hexachlorophene	70-30-4	5.98	405.90			0.4						
17	flame retardant	2,2',4,4',5-Pentabromodiphenyl ether	60348-60-9	7.29	564.69			14						
25	flame retardant	3,3',5,5'-Tetrabromobisphenol A	79-94-7	5.21	541.86			1.2						
7	drug/drug-like	6-Hydroxydopamine hydrochloride	28094-15-7	-0.41	170.19			2.4						
12	pesticide	Dieldrin	60-57-1	4.48	380.91			7.9						
65	flame retardant	2,2',4,4',5,5'-Hexabromodiphenyl ether	68631-49-2	8.05	643.59	°	°	18	°	not tested				
18	flame retardant	2,2'4,4'-Tetrabromodiphenyl ether	5436-43-1	6.53	485.80			3.5						
31	pesticide	Dichlorodiphenyltrichloroethane (DDT)	50-29-3	6.50	354.49			3.3						
45	pesticide	Heptachlor	76-44-8	5.24	373.32			4.6						
74	flame retardant	Phenol, isopropylated, phosphate (3:1)	68937-41-7	8.70	452.53			6.1						
11	plasticizer	2-ethylhexyl diphenyl phosphate (EHDP)	1241-94-7	6.49	362.41			4.5						
34	flame retardant	Tricresyl phosphate	1330-78-5	6.26	368.37			7.8						
61	pesticide	Chlorpyrifos (Dursban)	2921-88-2	4.72	350.59			12						
73	flame retardant	Isodecyl diphenyl phosphate	29761-21-5	7.27	390.46			11						
75	flame retardant	tert-Butylphenyl diphenyl phosphate	56803-37-3	6.63	382.40			4.5						
32	flame retardant	Triphenyl phosphate	115-86-6	5.33	326.29	*		10						
40	PAH	Acenaphthylene	208-96-8	3.32	152.20		*			#				
58	pesticide	Tebuconazole	107534-96-3	3.34	307.83	*								
8	drug/drug-like	Valproic acid sodium salt	1069-66-5	0.95	143.21									
48	industrial chemical	Manganese, tricarbonyl…	12108-13-3		218.09									
20	industrial chemical	1-ethyl-3-methylimidazolium diethylphosphate	848641-69-0		264.26									
15	plasticizer	Di(2-ethylhexyl) phthalate	117-81-7	6.43	390.56									
64	drug/drug-like	Diazepam	439-14-5	3.15	284.75									
30	pesticide	Deltamethrin	52918-63-5	6.49	505.21									
50	pesticide	Parathion	56-38-2	3.27	291.27					#				
36	pesticide	Permethrin	52645-53-1	6.11	391.29									
23	solvent	n-Hexane	110-54-3	2.59	86.18					#				
55	PAH	Fluorene	86-73-7	3.26	166.22									
38	PAH	Benzo(e)pyrene	192-97-2	5.74	252.32					#				
49	PAH	Naphthalene	91-20-3	2.84	128.17									
16	plasticizer	Bisphenol A	80-05-7	3.42	228.29									
13	PAH	Pyrene	129-00-0	4.58	202.26									
51	PAH	Benz(a)anthracene	56-55-3	5.15	228.29					#				
26	solvent	Toluene	108-88-3	2.00	92.14									
27	flame retardant	Tris(2-chloroethyl) phosphate	115-96-8	2.86	285.49									
24	PAH	4-H-Cyclopenta(d,e,f)phenanthrene	203-64-5	3.90	190.25									
62	PAH	Acenaphthene	83-32-9	2.94	154.21									
37	PAH	Phenanthrene	85-01-8	3.99	178.23									
56	PAH	Anthracene	120-12-7	3.99	178.23					#				
60	PAH	Benzo(b)fluoranthene	205-99-2	5.64	252.32					#				
22	PAH	Benzo(k)fluoranthene	207-08-9	5.64	252.32					#				
52	pesticide	Lindane	58-89-9	3.64	290.83									
54	food additive	L-Ascorbic acid	50-81-7	-3.80	174.11									
14	drug/drug-like	Tetraethylthiuram disulfide	97-77-8	3.62	296.55									
1	drug/drug-like	Hydroxyurea	127-07-1	-0.96	76.06									
3	industrial chemical	3,3'-Iminodipropionitrile	111-94-4	0.40	123.16									
43	industrial chemical	Acetic acid, manganese(2+) salt	638-38-0		173.03									
9	solvent	2-Methyoxyethanol	109-86-4	-0.38	76.10									
28	pesticide	Captan	133-06-2	2.91	300.59									
21	PAH	Benzo(a)pyrene	50-32-8	5.74	252.32									
39	drug/drug-like	6-Propyl-2-thiouracil	51-52-5	1.39	170.24									
47	food additive	D-Glucitol	50-70-4	-3.59	182.17									
10	drug/drug-like	Phenobarbital sodium salt	57-30-7	0.70	232.24									
53	drug/drug-like	Acetylsalicylic acid	50-78-2	-0.03	179.15									
57	pesticide	Aldicarb	116-06-3	1.47	190.27									
46	drug/drug-like	Acetaminophen (4-hydroxyacetanilide)	103-90-2	1.35	151.17									
71	PAH	Dibenz[a,c]anthracene	215-58-7	6.30	278.35									
6	food additive	Saccharin Sodium Salt hydrate	82385-42-0	0.80	182.18									
4	drug/drug-like	Thalidomide	50-35-1	0.09	258.23					#				
59	drug/drug-like	5-Fluorouracil	51-21-8	-0.80	130.08									
70	PAH	Benzo[g,h,i]perylene	191-24-2	6.33	276.34	°	°	°	°	not tested				
66	PAH	Chrysene	218-01-9	5.15	228.29	°	°	°	°	not tested				
67	PAH	Dibenz(a,h)anthracene	53-70-3	6.30	278.35	°	°	°	°	not tested				
69	env. t./heavy metal	Bis(tributyltin)oxide	56-35-9		596.10	°	°	°	°	not tested				
72	env. t./heavy metal	2,3,7,8-Tetrachlorodibenzo-p-dioxin	1746-01-6	6.20	321.97	°	°	°	°	not tested				

specific | cytotoxic | no effect

Fig. S1: Cross-comparison of test data for the NTP80 collection
NeuriTox (= UKN4) and PeriTox (= UKN5) data obtained here are shown in the context of published data from other studies on the NTP80 collection. The effect of the chemicals on the different tests is indicated as specific effect on cell function (blue), cytotoxic effect (red), or no effect (white); light red coloring indicates that the used assay did not discriminate between specific effects and cytotoxicity ([4] = Pei et al., 2016). For the specific hits of the UKN4, UKN5, and UKN2 (= cMINC: [1] = Nyffeler et al., 2017a) tests,

This is an Open Access article distributed under the terms of the Creative Commons Attribution 4.0 International license (http://creativecommons.org/licenses/by/4.0/), which permits unrestricted use, distribution and reproduction in any medium, provided the original work is appropriately cited.

doi:10.14573/altex.1712182s1

the EC_{25} value for the most sensitive endpoint is given in µM. For the NeuriTox test, specific hits were defined by an EC50(V/NA) ratio of ≥ 4, for the PeriTox test the ratio had to be ≥ 3. For the cMINC test, compounds inhibiting migration ≥ 25% without affecting viability by more than 10% were considered specific. For the alternative neurite outgrowth model ([2] = Ryan et al., 2016), specificity was defined as ratio between BMC concentrations for viability and neurite area ≥ 3.16 and the confirmation of this classification in a retesting. In the cardiotoxicity test ([3] =Sirenko et al., 2017), substances were defined as specific if they i) affected cardio-physiologic parameters after 30 min treatment at a three-fold lower concentration than viability and ii) if they affected viability by < 10% after 24 h. If not stated otherwise, NeuriTox, PeriTox, and cMINC were performed with 20 µM as highest concentration, with a DMSO concentration of 0.1%. Other assays were performed at concentrations up to 100 µM (with up to 0.5% DMSO in the test). Compound number in the NTP80 collection, compound classification, Chemical Abstracts Service Registry Number (CAS#), molecular weight, and hydrophobicity (logP) are given as additional information. An asterisk (*) indicates that the substance was tested at higher than standard concentrations, ° indicates that a substance was tested at lower than standard concentrations, # indicates that the calcein signal was impaired, but the authors did not conclude cytotoxicity from that. Abbreviations: PAH = polycyclic aromatic hydrocarbon, env. t./heavy metal = environmental toxicant/heavy metal.

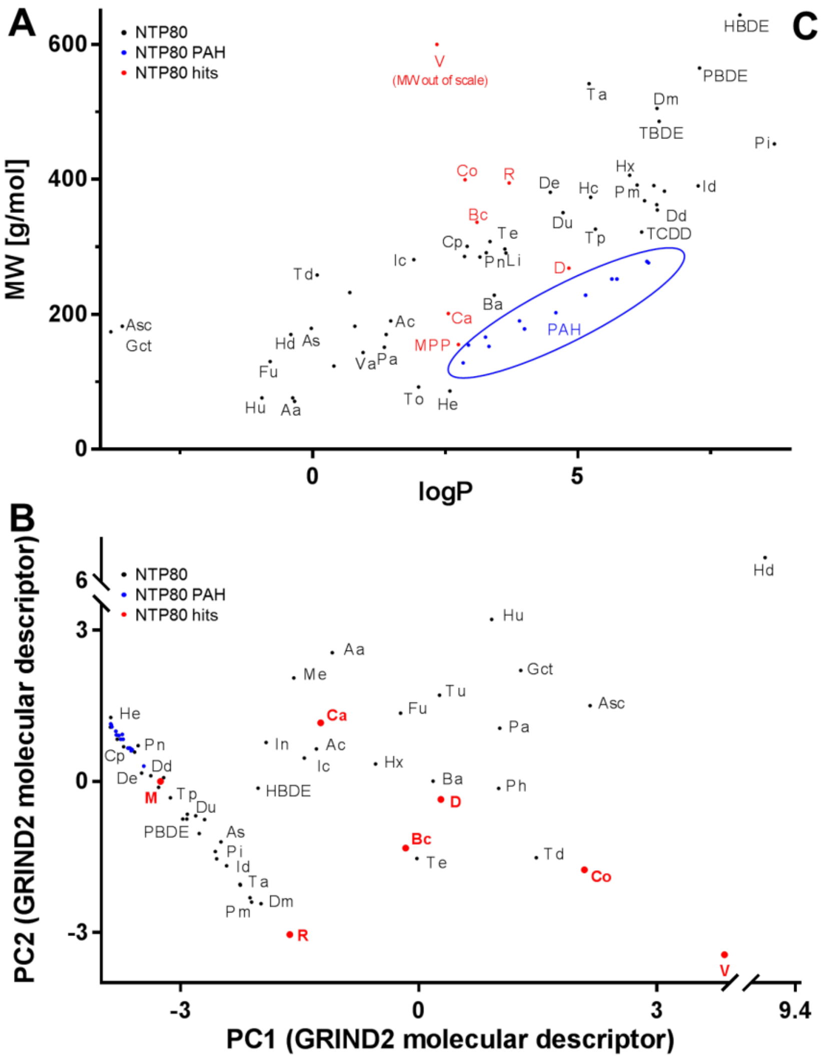

C

Abbr.	*Chemical name*
Aa	Acrylamide
Ac	Aldicarb
As	Acetylsalicylic acid
Asc	L-Ascorbic acid
Ba	Bisophenol A
Bc	Berberine chloride
Ca	Carbaryl
Co	Colchicine
Cp	Captan
D	Diethylstilbestrol
Dd	DDT
De	Dieldrin
Dm	Deltamethrin
Du	Chlorpyrifos (Dursban)
Fu	Fluoruracil
Gct	Glucitol
HBDE	2,2,4,4,5,5-Hexabromodiphenylether
Hc	Heptachlor
Hd	Hydroxydopamine
He	n-Hexane
Hu	Hydroxyurea
Hx	Hexachlorophen
Ic	Iodocarb
Id	Isodecyl diphenyl phosphate
In	3,3'-Iminodipropionitrile
Li	Lindane
Me	2-Methyoxyethanol
M	1-Methyl-4-phenylpyridinium
Pa	Acetaminophen
PAH	Polycyclic aromatic hydrocarbon
PBDE	2,2,4,4,5-Pentabromodiphenylether
Ph	Phenobarbital
Pi	Phenol isoprpylated, phosphate 3:1
Pm	Permethrin
Pn	Parathione
R	Rotenone
Ta	3,3,5,5-Tetrabrombisphenol A
TBDE	2,2,4,4-Tetrabromodiphenylether
TCDD	2,3,7,8-Tetrachlorodibenzo-p-dioxin
Td	Thalidomide
Te	Tebuconazole
To	Toluene
Tp	Triphenylphosphate
Tu	6-Propyl-2-thiouracil
V	Valinomycin
Va	Valproic acid

Fig. S2: Characterization of the chemical properties of the screened library
A) Characterization of the hydrophobicity and molecular weight of the NTP80 collection chemicals. Coloring was chosen for polycyclic aromatic hydrocarbons (PAH, blue), which cluster closely together in the lower right part of the plot, and for chemicals that were identified as hits in the NeuriTox test (red). Hit substances covered the full range of molecular weights and they were in the hydrophobicity range between logP 2 and logP 5. B) An extensive set of molecular descriptors was generated for the combined Tox21 and DrugBank libraries as well as for the NTP80 collection. The chemical descriptors of the Tox21 and DrugBank libraries were used to define the axes of the principal component analysis in which the chemicals of the NTP80 collection were plotted. The specific hits of the screen described later in this publication are highlighted in red – they are spread over the complete described chemical space. C) Table of abbreviations of chemicals of the NTP80 collection.

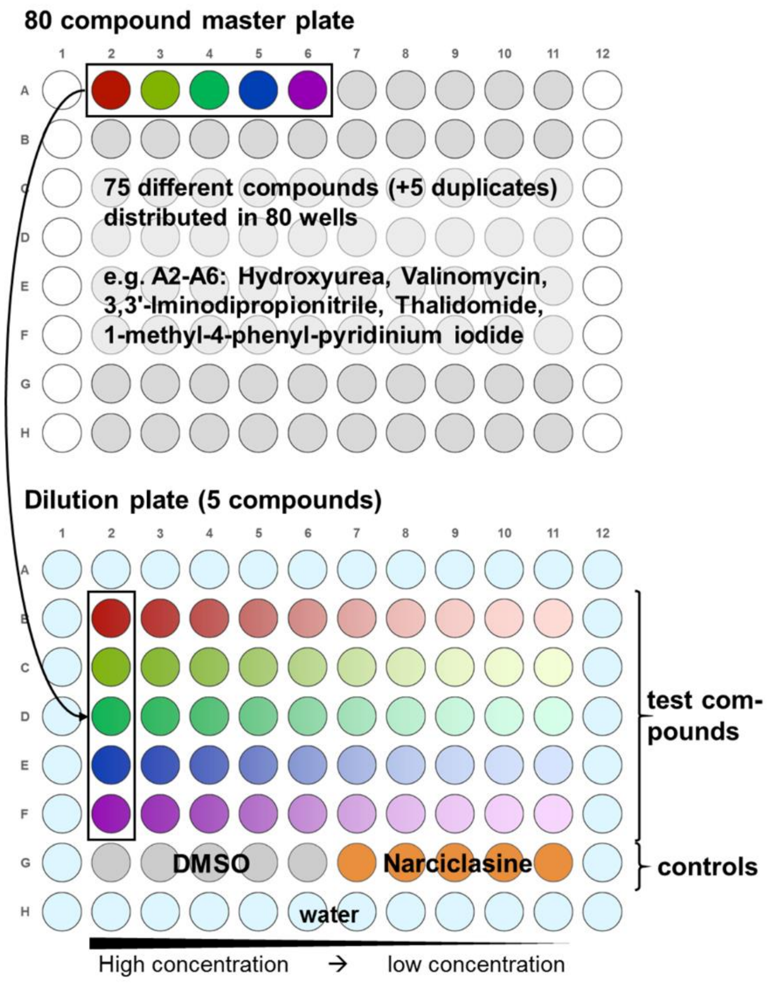

Suppl. Fig. S3: Chemical library handling
The "master plate" of chemicals was delivered with each well (A2-H11) filled with a different chemical (75 unique chemicals plus 5 internal duplicates as control). To keep the number of freeze-thaw cycles low and equal between the experiments, five compounds from the "master plate" were always transferred to the "dilution plate", where they were diluted in DMSO and complemented with DMSO wells (solvent control) and narciclasine (50 µM on "dilution plate", 50 nM final concentration on cells, positive control). Several copies (= "aliquots") of the "dilution plates" were produced and stored at -80°C until use.

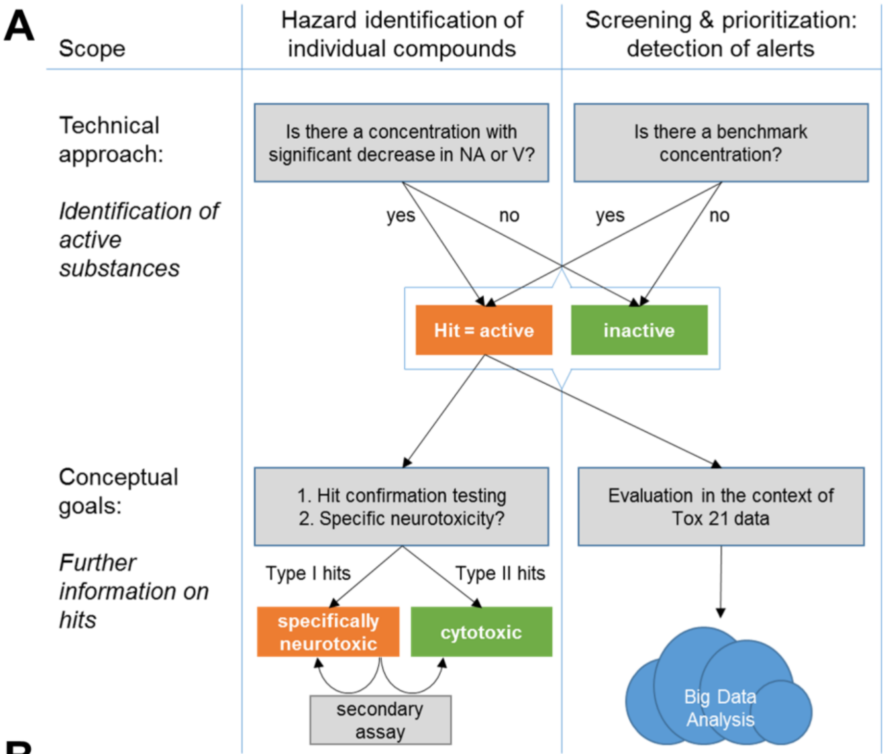

B

compound \ method	EC50 [µM] from screen + hit confirmation data: Neurites	Viability	ratio V/NA	spec.	BMC [µM] from screen data: Neurites	Viability	ratio V/NA	spec.	Classi-fication
Valinomycin	0.01	6.2	616	s	0.3	0.5	2.0	c	Hit(N)
Colchicine	0.03	10.0	333	s	0.01	0.1	9.0	s	Hit
Methylmercury (II) chloride	0.09	0.2	1.8	c	0.1	0.1	1.7	c	&
Bis(tributyltin)oxide	0.3	0.4	1.2	c	0.2	0.2	1.3	c	&
Rotenone	0.5	40.0	80	s	0.03	20.0	670	s	Hit
Iodocarb	0.9	1.2	1.3	c	0.4	0.2	0.4	c	&
Captan	2.7	4.3	1.6	c	16.7	20.0	1.2	c	&
TETD	3.7	2.9	0.8	c	3.0	2.5	0.8	c	&
Hexachlorophene	7.8	18.3	2.3	c	7.1	12.7	1.8	c	&
Diethylstilbestrol	8.9	36.7	4.1	s	2.6	20.0	7.8	s	Hit
Berberine chloride	23.9	126.0	5.3	s	4.7	20.0	4.3	s	Hit
Carbaryl	30.9	132.1	4.3	s	8.5	20.0	2.3	c	Hit(N)
MPP+	80.0	320.0	4.0	s	1.3	20.0	15	s	Hit
Tebuconazole	142.0	238.0	1.7	c	>20	>20	1.0	n.d.	$
Bisphenol A	>20	>20	1.0	n.d.	0.4	0.2	0.4	c	*
2,2',4,4',5-PBDE	>20	>20	1.0	c	8.1	2.3	0.3	c	°
2,2'4,4'-TBDE	>20	>20	1.0	c	20.0	1.0	0.1	c	°

Fig. S4: Different scopes of data usage determine strategies for hit compound definitions
A) Different data usages demand alternative data evaluation procedures. For hazard identification of compounds or for quantitative follow-up (e.g., QSAR, read-across), screen hits have to be confirmed by detailed hit confirmation testing in the same assay. Moreover, confirmation in a secondary assay is desirable. For initial screening and prioritization, in the context of big data sets like Tox21, priorities are rather on rapid processing and comparability of the hit definitions across multiple diverse assays.
B) Comparison of the interpretation of NeuriTox test outcomes by either the EC_{50} method (NeuriTox prediction model), or the BMC method. Substances that were found to be active by at least one of the methods are shown. The specificity threshold for the NeuriTox (and PeriTox) test was originally derived by statistical values from noise data in the assays (specific effect if EC_{50} ratio V/NA ≥ 4, Fig. 3). The transformation of an EC_{50} ratio into a BMC ratio at about the EC_{20} level is not trivial. Curve variability, curve steepness, and steepness-dependence of variability differ in different parts of a fitted curve. Thus, here a BMC ratio of ≥ 3.16 was used as selectivity threshold, as this value has been used in the literature (Ryan et al., 2016).
Substance classification: c= cytotoxic, s= specific hit. HIT: classified by both methods as specific hit. &: classified by both methods as cytotoxic. HIT(N): classified as active by both methods. Cytotoxic according to BMC method, specific according to NeuriTox (N) method. $: retested and classified as cytotoxic by N method; no BMC derivable. Concentrations are outside the screen range. *: BMC was derived from screen data and thus classified as cytotoxic. Retesting and EC_{50} analysis revealed no toxicity in the range up to 20 µM. °: Classified as active but cytotoxic by EC_{50} method but with EC values > 20 µM. 2,2',4,4',5-PBDE: 2,2',4,4',5-pentabromdiphenylether, 2,2'4,4'-TBDE: 2,2'4,4'- tetrabromdiphenylether.

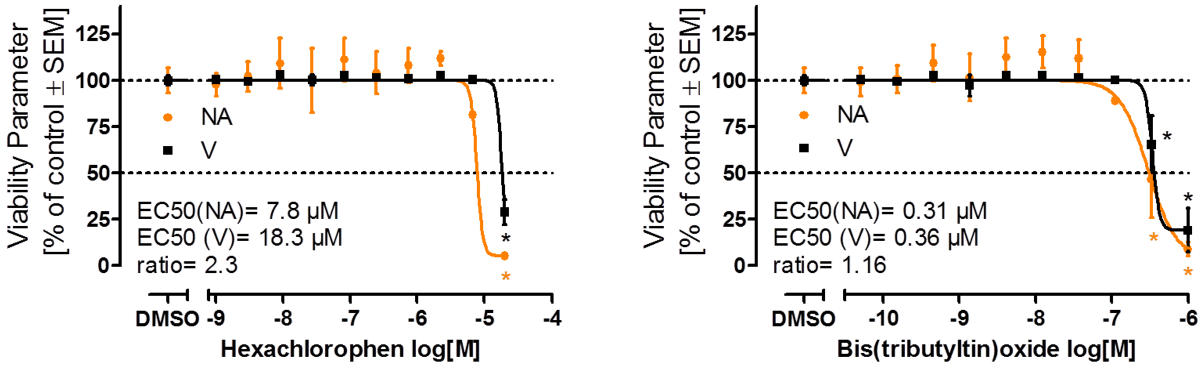

Suppl. Fig. S5: Examples of NeuriTox screen data for cytotoxic substances
A) LUHMES cells, differentiated for two days, were plated at a density of 100,000 cells/cm² into 96-well plates, treated one hour later, and analyzed after 24 h. Neurite area (NA, orange) and viability (V, black) were determined by high content imaging. Concentration-response curves are given for two representative cytotoxic substances (hexachlorophen and bis(tributyltin)oxide). Since these substances were clearly cytotoxic, EC_{50} values were calculated directly from the screen results without re-testing. All data are means ± SEM from three biological replicates, dashed lines are drawn at 100% and 50%. *: $p < 0.05$, by one-way ANOVA followed by Dunnett's post-hoc test.

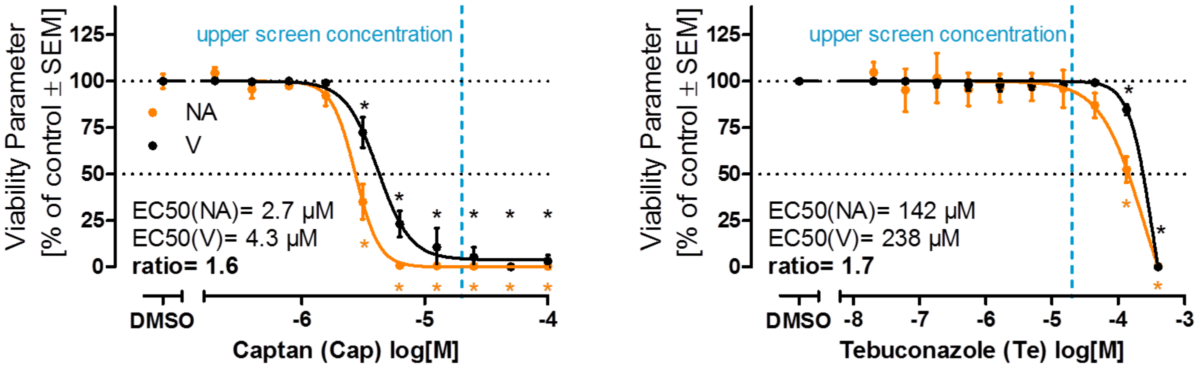

Suppl. Fig. S6: Hit confirmation testing in NeuriTox test of substances that were subsequently classified as unspecific cytotoxicants
A) Substances that were classified as hits after the first round of screening were re-ordered independently and re-tested for their effect on neurite outgrowth inhibition (NA, orange) and viability (V, black) in an adjusted concentration range (otherwise same experimental setup as for the screening). EC values and their ratios were calculated from four-parameter log-logistic fit functions. The substances were classified as unspecific cytotoxic chemicals (EC_{50} ratio (V/NA) < 4). For orientation, the highest original screen concentration (20 µM) is indicated (blue dashed line). All data are means ± SEM from three biological replicates, dashed lines are drawn at 100% and 50%. *: $p < 0.05$, by one-way ANOVA followed by Dunnett's post-hoc test.

A

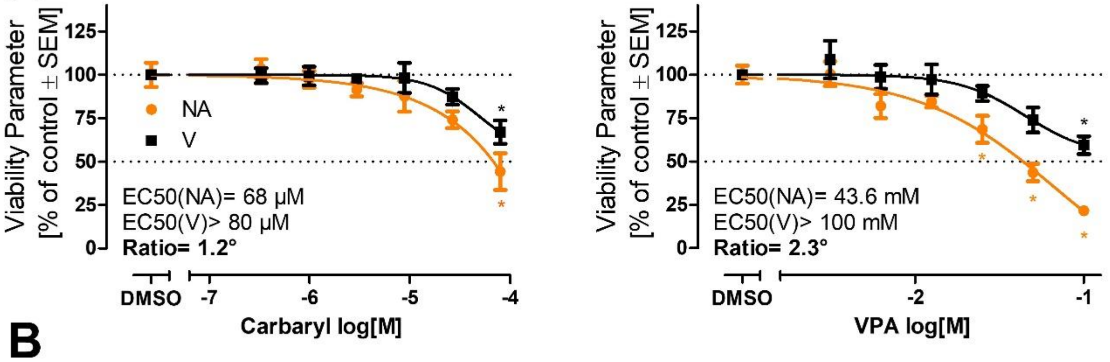

B

Data in [µM]	Carbaryl	VPA
Ratio(EC30)	2.1	2.6
Ratio(EC25)	2.6	2.7
Ratio(EC20)	2.3	2.9
Ratio(BMC)	0.7	2.0

Fig. S7: Comparison of different BMC and EC values and ratios for the PeriTox test, for example substances that were classified as unspecific cytotoxicants

A) Substances that were classified as hits after the first round of screening were re-ordered independently and re-tested for their effect on neurite outgrowth inhibition (NA, orange) and viability (V, black) in an adjusted concentration range (otherwise same experimental setup as for the screening). EC values and their ratios were calculated from four-parameter log-logistic fit functions. B) EC ratios (V/NA) in the low toxicity range (EC_{20}, EC_{25} or EC_{30}) are all < 3 and thus do not classify the compounds as hits. The BMC values were calculated for the benchmark responses of 3x SD of control cells, as used by the NTP. The BMC ratio was < 3, thus supporting our classification of the compounds as non-specific toxicants.

All data are means ± SEM from three biological replicates, dashed lines are drawn at 100% and 50%. *: $p < 0.05$, by one-way ANOVA followed by Dunnett's post-hoc test.

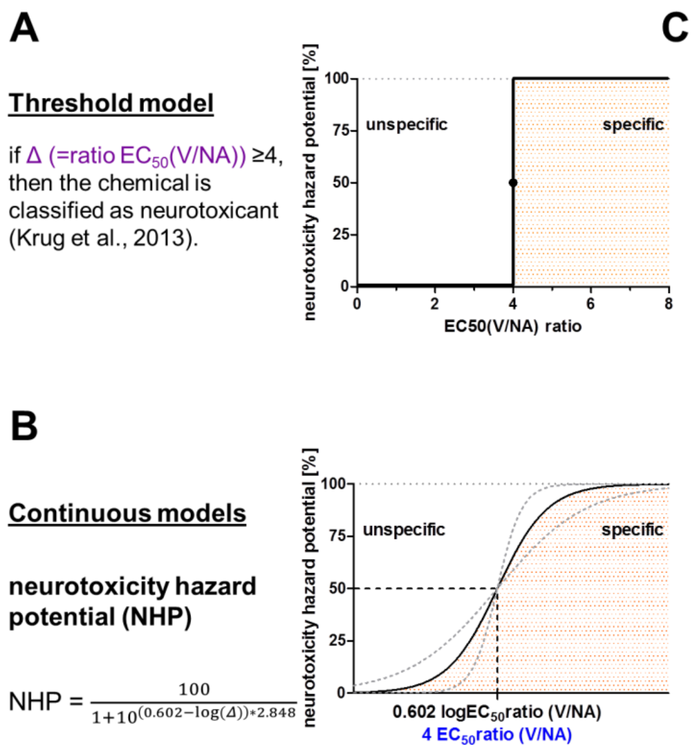

C

classification / compound	EC50 ratio	Threshold model [hit/no hit]	NHP [% hit probability]
Tetraethyl-thiuram disulfid	0.8	no hit	1
Bis(tributyltin) oxide	1.2	no hit	3
Iodocarb	1.3	no hit	4
Captan	1.6	no hit	7
Tebuconazole	1.7	no hit	8
Methylmercury(II) chloride	1.8	no hit	9
Hexachlorophen	2.3	no hit	17
Diethylstilbestrol	4.1	hit	52
Carbaryl	4.2	hit	53
Berberine chloride	5.3	hit	69
Rotenone	20	hit	99
MPP+	83	hit	100
Colchicine	333	hit	100
Valinomycin	620	hit	100

Suppl. Fig. S8: Exemplification of one possible hazard potential model with few example data on NeuriTox hits/non-hits
A) Graphical illustration of the fixed threshold prediction model. For values of $\Delta < 4$, compounds are classified as unspecific toxicants, while $\Delta \geq 4$ is assumed to indicate a specific neurotoxicant. B) A set of potential curves are displayed that can relate Δ to the hazard probability. The variation of Δ (SD(Δ)) was calculated from a large number of experiments and used to parametrize a calibration curve for a continuous prediction model of the NeuriTox assay. It is based on SD(Δ) = 0.254, and assumes a log-logistic curve with upper and lower asymptotes = 0% and 100% for the transition. C) Using the curve formula displayed in B, the curve offsets (V vs NA = Δ) for various compounds measured in this study were converted to hazard probabilities. Compounds indicated in orange refer to the NeuriTox hit compounds; blue indicates compounds that had been found to be non-specific toxicants.
